# Capacitive deionisation for water desalination review: experimental and simulation

**DOI:** 10.1080/14686996.2025.2546286

**Published:** 2025-08-19

**Authors:** Rokhsareh Akbarzadeh, Mathias Ernst, Robert Meißner, Bodo Fiedler

**Affiliations:** a Institute of Polymers and Composites, Hamburg University of Technology, Hamburg, Germany; b Institute for Water Resources and Water Supply, Hamburg University of Technology, Hamburg, Germany; c Institute of Soft Matter Modeling, Hamburg University of Technology, Hamburg, Germany; d Institute of Surface Science, Helmholtz-Zentrum Hereon, Geesthacht, Germany

**Keywords:** Capacitive deionisation (CDI), desalination, electrosorption, MCDI, FCDI, simulation, ion removal

## Abstract

Capacitive Deionization (CDI) has emerged as an energy-efficient and environmentally friendly technology for water desalination. This review provides a comprehensive analysis of CDI, covering both experimental and simulation approaches. It introduces the background, definition, and diverse applications of CDI, from water desalination to environmental monitoring and resource recovery. The review highlights CDI’s advantages, such as low energy consumption and operational simplicity, as well as its limitations, particularly its design-specific operating window favoring low-to-moderate salinity waters and sensitivity to organic-rich conditions. Strategies such as hybrid CDI systems and electrode surface functionalization are discussed to mitigate these challenges. Key working principles and advancements, including innovations in electrode materials, synthesis methods, and reactor design, are examined to improve ion removal efficiency, selectivity, energy use, and system durability. Material modification strategies are presented in the context of structure – performance relationships, emphasizing rational design principles. The review also explores simulation methods, including reactor modeling, computational fluid dynamics, molecular dynamics, and numerical approaches, and machine learning highlighting their synergy with experiments in optimizing CDI performance and guiding scale-up. Coupling CDI with other systems and its applications in water purification, particularly for ion and organic compound removal are also discussed. Finally, challenges in both experimental and simulation efforts, such as material cost, model complexity, computational demands, and scalability, are discussed. While CDI shows promise for sustainable water desalination and resource recovery, further research on hybrid configurations, predictive modeling, and pilot-scale validation is needed to address its limitations and enable large-scale adoption.

## IMPACT STATEMENT

This review uniquely integrates experimental advances and simulation techniques in CDI, providing a unified perspective to guide research and practical implementation for sustainable water desalination.

## GRAPHICAL ABSTRACT

**Figure:**
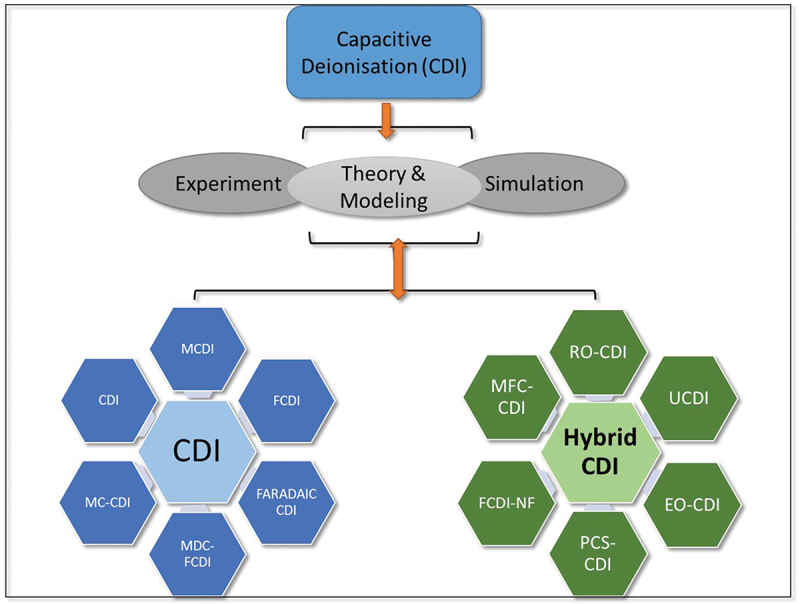

## Introduction

The scarcity of clean and potable water has become a pressing global issue, exacerbated by rapid economic development, agricultural expansion, and population growth. Severe water pollution from industrial, agricultural, and domestic sources further intensifies the freshwater crisis, making effective water desalination technologies crucial for sustainable water resource management. Traditional desalination methods such as multi-stage flash, multi-effect distillation, vapor compression, and electro-dialysis, while effective, are often constrained by high energy demands, significant costs, and environmental impacts [1].

Capacitive Deionization (CDI) has emerged as a promising green desalination technology due to its energy efficiency, simplicity, and environmentally friendly process. CDI operates based on the principle of ion electrosorption at the surface of electrically charged electrodes, typically composed of activated carbon or other high surface area materials. The technology offers significant advantages over conventional methods, including reduced energy consumption, minimal chemical usage, and cost-effectiveness, making it suitable for treating low-salinity waters such as brackish water [2,3].

The origins of CDI can be traced back to M. Faraday’s electrochemical discoveries in 1833 [4], with the technology seeing substantial advancements over the past decades, notably with the introduction of carbon aerogels by J. Farmer in the 1990s [5]. These materials have greatly improved the adsorption capacities and performance of CDI systems [6]. More recently, innovations like Membrane Capacitive Deionization (MCDI) and Flow-Electrode Capacitive Deionization (FCDI) have further enhanced CDI’s capabilities by integrating ion-exchange membranes and flowable electrodes, leading to improved charge efficiency, selectivity, and continuous operation in challenging conditions [7,8]. The growing interest in CDI technology is evidenced by the exponential increase in publications and patents over the past few decades, particularly highlighting its potential in sustainable water desalination and resource recovery [9].

Despite these advancements, CDI still faces challenges including electrode fouling, material durability, and the need for standardized testing protocols [3,7]. Material innovations, particularly in carbon-based electrodes such as graphene, carbon nanotubes, and carbon aerogels, have been critical to improving ion adsorption capacities and overall CDI performance. However, the high cost of these materials and the need for scalable designs remain significant hurdles [3,9].

This review provides a comprehensive overview of recent advancements in CDI technology, integrating both experimental and simulation approaches. It addresses the development of electrode materials, reactor design, and working principles and advancements to optimize ion removal efficiency, energy use, and system durability. Furthermore, this paper highlights the critical role of simulation methods such as reactor modeling, computational fluid dynamics (CFD), molecular dynamics, and numerical modeling in enhancing CDI performance and aligning experimental efforts with theoretical predictions.

While earlier reviews have focused on specific aspects of CDI such as electrode materials, operational principles, or reactor configurations [2,3,7,10–12], or adopted a somewhat broader scope such as the work by Miao et al. [13], which extended the discussion to methodological advances but remained centered on CDI adsorption mechanisms. The present review provides a truly comprehensive and application-oriented examination of CDI as a water desalination technology. It goes beyond these previous perspectives by discussing recent advances in electrode materials, reactor architectures, operational strategies, hybrid and coupled systems, and the key performance challenges and possible solutions, drawing on both experimental and simulation studies. This broader perspective offers valuable insights not only into the fundamental understanding of CDI processes but also into practical pathways for enhancing performance, scalability, and applicability of CDI for sustainable water treatment. In doing so, this review bridges the gap between simulation and experimental methodologies, helping to advance CDI research from concept to implementation.

### Background and definition of CDI and its terms

**Figure 1.**
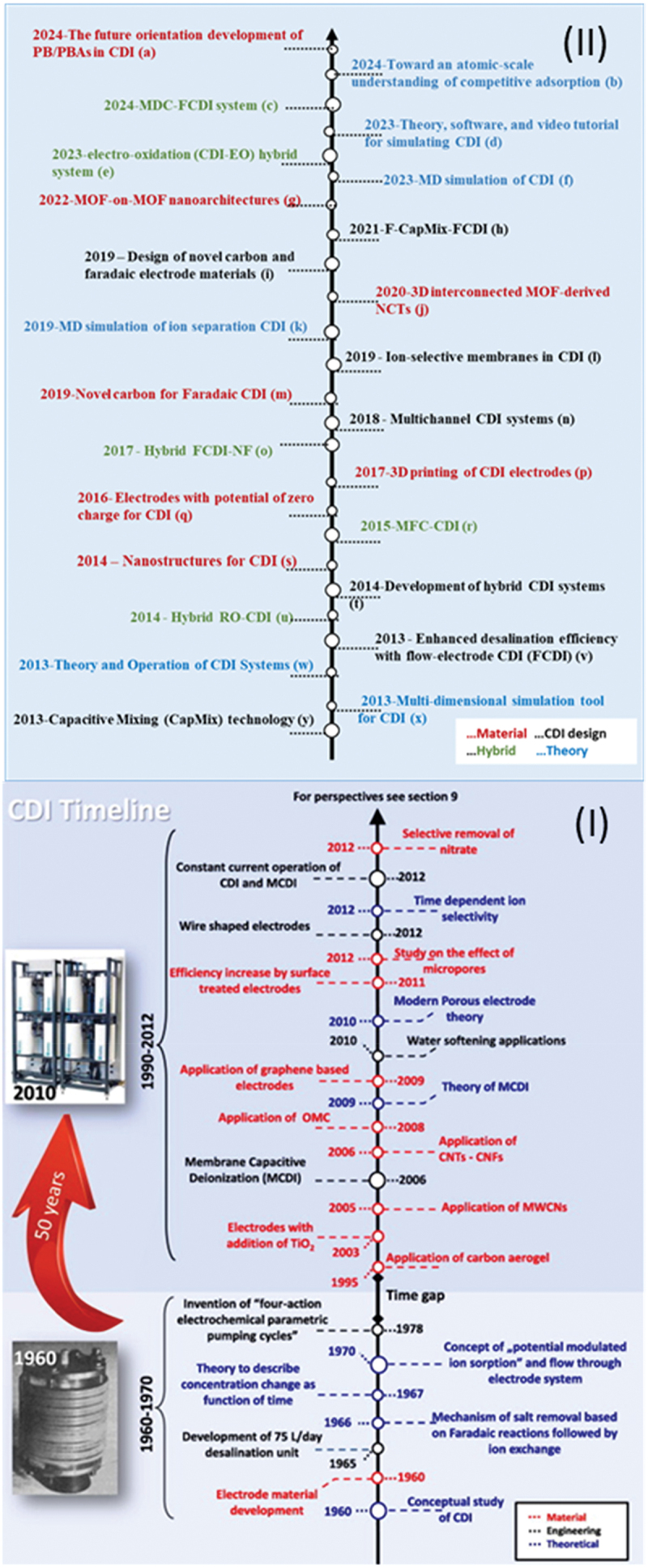
(I) Timeline of scientific developments of CDI, indicating milestones since the inception of CDI in 1960 [11]. and timeline since 2013 up to 2024 a) [15], b) [16], c) [17], d) [18], e) [19], f) [20], g) [21,22], h) [23,24], i) [25], j) [26], k) [27], l) [28], m) [29], n) [30], o) [31], p) [32], q) [33] r) [34], s) [35], t) [36], u) [37], v) [38], w) [38], x) [39,40], y) [41], reproduced by permission from Ref. [11] obtained via copyright clearance center.

CDI has gained recognition due to its energy efficiency and suitability for brackish water desalination. The technology, rooted in electrochemical capacitor principles, has been widely studied as an alternative to conventional desalination processes such as reverse osmosis [14]. A foundational review by Oren (2008) critically assessed the early CDI landscape, identifying challenges such as low charge efficiency, limited salt adsorption capacity, electrode fouling, and energy losses during regeneration. These limitations underscored the need for innovation in electrode materials, system design, and operational strategies. Building upon this early critique, a more comprehensive historical review by Porada et al. outlined the evolution of CDI technologies and key milestones from 1960 to 2012 [11]. In Figure 1 (I) they illustrate this timeline, marking the progression of CDI research and system innovation over five decades.

Over the decades, advances in materials science, particularly the development of high-surface-area carbon materials like carbon nanotubes and graphene, have significantly enhanced the performance and applicability of CDI systems. Innovations such as Membrane CDI (MCDI) and Flow-Electrode CDI (FCDI) have further expanded the versatility and efficiency of this desalination technology.

To extend this historical perspective, we added Figure 1 (II) to presents a continuation of the CDI timeline from 2013 to 2024, highlighting key developments in four major categories: materials, CDI system design, hybrid configurations, and theoretical advancements. This updated literature timeline captures the recent surge in research exploring MOF-based architectures, hybrid CDI-RO or MFC systems, nanostructured electrode materials, and advanced modeling techniques including 3D and MD simulations. Notably, recent years have also seen the emergence of integrated CDI concepts like electro-oxidation-CDI hybrids, as well as efforts toward atomic-scale understanding of competitive ion adsorption. By bridging past progress with ongoing innovation, the combined timeline illustrates the rapidly diversifying landscape of CDI research and its move toward more multifunctional, efficient, and application-specific solutions.

#### Key terms in CDI technology

**Figure 2.**
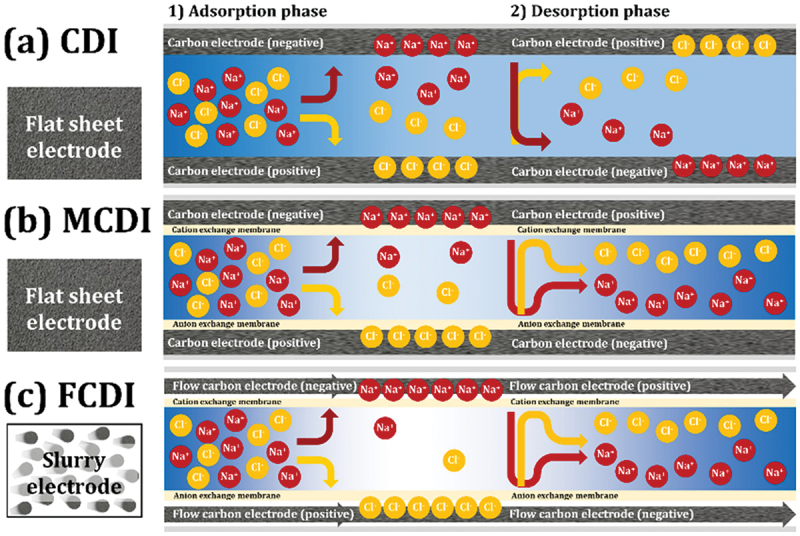
Removal mechanisms of (a) CDI, (b) membrane-CDI, and (c) flow electrode-CDI [25]. Reproduced with permission obtained via copyright clearance center.
- Electrode: The components within the CDI cell where the ions are adsorbed onto the electrode surface through electrostatic forces. In CDI, electrodes are typically made from carbon-based materials with high surface areas, such as activated carbon, carbon aerogels, graphene oxide or graphene. The electrodes’ primary role is to adsorb ions from the water during the desalination process.

- CDI: Capacitive Deionization (CDI) is an electrochemical process used for desalinating water by removing ions. The process involves applying an electrical potential difference across a pair of porous electrodes, which leads to the migration and adsorption of charged ions (cations and anions) from the water onto the surface of the electrodes. As shown in Figure 2a, when a voltage is applied, cations move toward the negatively charged electrode, while anions move toward the positively charged electrode, effectively reducing the salt content in the water. Once the electrodes are saturated with ions, the system can be regenerated by reversing the voltage or short-circuiting the electrodes, releasing the ions into a waste stream.

#### Types of electrodes

- Negatively charged electrode: The negatively charged electrode that attracts cations (positively charged ions).
- Positively charged electrode: The positively charged electrode that attracts anions (negatively charged ions).

- Electrical Double Layer (EDL): The solid-liquid structure formed at the interface between the electrode surface and the electrolyte (water) in CDI systems. Although limited in its applicability, the well-known Stern model, which consists of a layer of ions adsorbed on the electrode surface and a diffuse layer of oppositely charged ions in the water, is often used to describe the EDL on a theoretical basis. The formation of the EDL is essential for the ion adsorption process in CDI.
- Ion-Exchange Membrane: A membrane that selectively allows certain ions to pass through while blocking others. In Membrane Capacitive Deionization (MCDI), ion-exchange membranes are used in conjunction with electrodes to enhance the selectivity and efficiency of the ion removal process. Cation-exchange membranes allow only cations to pass, while anion-exchange membranes allow only anions to pass.
- Membrane Capacitive Deionization (MCDI): An advanced form of CDI where ion-exchange membranes are placed in front of the electrodes. The membranes enhance charge efficiency by preventing the co-ions (ions of the same charge as the electrode) from being adsorbed onto the electrodes, thus improving the overall desalination performance and energy efficiency. The bottom part of Figure 2b illustrates Membrane Capacitive Deionization (MCDI), an advanced form of CDI that incorporates ion-exchange membranes. In MCDI, a cation-exchange membrane is placed in front of the negatively charged electrode, allowing only cations to pass through, while an anion-exchange membrane is placed in front of the positively charged electrode, allowing only anions to pass. As a result, MCDI improves the overall desalination performance and energy efficiency compared to standard CDI by enhancing selectivity and minimizing the loss of electrochemical capacity to co-ion adsorption.
- Flow-Electrode Capacitive Deionization (FCDI): A variant of CDI where the electrodes are not stationary but are instead a slurry of conductive particles (often carbon-based) suspended in an electrolyte. This flowable electrode system allows for continuous operation and easier scaling of the CDI process, particularly for higher salinity waters, as it is illustrated in Figure 2c.
- Multiple channels CDI (MC-CDI): A capacitive deionization (CDI) system architecture that employs multiple channels separated by capacitive membrane electrodes (CMEs). These CMEs act as selective membranes that allow targeted ion transport when charged, enhancing the system’s overall desalination efficiency. The design improves desalination performance by balancing low ionic resistance, for effective ion transfer, with permselectivity, to prevent unwanted ion migration.
- Desorption: The process by which adsorbed ions are released from the electrode surfaces during the regeneration phase of CDI. Desorption can be achieved by reversing the voltage applied to the electrodes or by short-circuiting them, allowing the ions to return to the water or a waste stream.
- Electrosorption Capacity: A measure of the mass of ions that can be adsorbed onto the electrodes per unit of electrode mass or surface area. High electrosorption capacity indicates more efficient ion removal in the CDI process.
- Cyclic Voltammogram (CV): An electrochemical measurement technique used to study the electrochemical properties of electrodes in CDI. CV plots the current that flows through the electrode material as a function of the applied voltage, providing insights into the capacitive behavior and ion adsorption capabilities of the electrode material.
- Regeneration Efficiency: The effectiveness with which a CDI system can release adsorbed ions during the desorption phase and restore the electrodes to their original state. High regeneration efficiency is critical for the continuous operation and longevity of the CDI system.
- Faradaic Reactions: Electrochemical reactions that involve the transfer of electrons between the electrode and ions in the electrolyte. While CDI is primarily a capacitive process, Faradaic reactions can occur, especially in hybrid systems or when using certain electrode materials, leading to oxidation or reduction reactions that may either enhance or hinder the performance of CDI.
- Electrode Fouling in CDI: Electrode fouling in Capacitive Deionization (CDI) refers to the accumulation of unwanted substances, such as organic compounds, microorganisms, or inorganic matter (e.g. calcium or magnesium salts), on the surface of the electrodes. This build-up interferes with the ion adsorption process by blocking active sites, reducing the electrochemical performance and efficiency of the CDI system. Fouling leads to decreased ion removal efficiency, higher energy consumption, and can shorten the lifespan of the electrodes. Effective fouling management and cleaning strategies are essential to maintain the long-term operational performance of CDI systems.
- Capacitance: Capacitance is the ability of a system, typically an electrical component like a capacitor, to store electrical charge. In the context of CDI, it refers to the electrode’s capacity to store ions within the electrical double layer formed at the electrode-electrolyte interface when a voltage is applied. Higher capacitance allows the electrodes to adsorb more ions, improving the efficiency of ion removal from water. While factors like surface area, porosity, and pore structure have traditionally been considered key influences on capacitance, recent findings suggest that structural disorder in carbon electrodes such as smaller, disordered graphene-like domains can significantly enhance capacitance [42]. This disorder creates more efficient ion storage within the nanopores, revealing a new approach to improving energy-dense systems for CDI.

### CDI operational mechanics and system innovation

The CDI process involves applying an electrical potential to a pair of electrodes to attract and remove ions from water. The process consists of two steps: adsorption (when ions are removed) and desorption (when electrodes are regenerated). This operation typically involves applying a differential voltage of between 1 and 1.4 V across porous electrodes [43]. This voltage drives the migration of salt ions to the electrical double layers formed at the electrode/electrolyte interfaces, effectively achieving desalination.

CDI is distinct from other electrochemical desalination technologies like Electrodialysis (ED). While ED uses ion-selective membranes and involves oxidation-reduction reactions as the primary mechanism, these faradaic reactions could also be used and could have potentially a great effect, but for now they are usually secondary to the capacitive process. This difference is crucial in determining the energy efficiency and scalability of CDI compared to ED [6].

Recent advancements have led to the development of a new CDI cell architecture with multiple channels (MC-CDI) where these channels separated by capacitive membrane electrodes (CMEs), which act as selective membranes when charged [44,45]. Studies on these CMEs revealed that those membrane electrodes with higher fluid permeability also demonstrated lower ionic resistances, thereby affecting their permselectivity and, consequently, the overall desalination performance. This insight underscores the need for CMEs to achieve a balance between low ionic resistance for effective ion transfer and adequate permselectivity to prevent undesired ion migration, optimizing the desalination performance in multi-channel CDI systems.

Furthermore, the continuous development of CDI technology has focused on enhancing the performance of membrane CDI (MCDI) and flow-electrode CDI (FCDI) systems. The introduction of hybrid CDI systems and integration of CDI with other desalination technologies have led to significant improvements in efficiency and cost-effectiveness, particularly in treating low to moderate salinity water [9].

According to a review [11], CDI has made significant progress with the use of porous carbon electrodes, which are fundamental in enhancing the energy efficiency of water desalination. The diversity of electrode materials, ranging from activated carbons to advanced graphene composites, is thoroughly evaluated for their salt adsorption efficiencies and stability [46]. Various CDI designs, such as standard dual-electrode setups and innovative membrane-assisted configurations, are discussed for their operational efficacy.

Figure 4 effectively illustrate the basic principles and setups of CDI, MCDI and FCDI, which is crucial to understand the operational fundamentals.

### Applications

**Figure 3.**
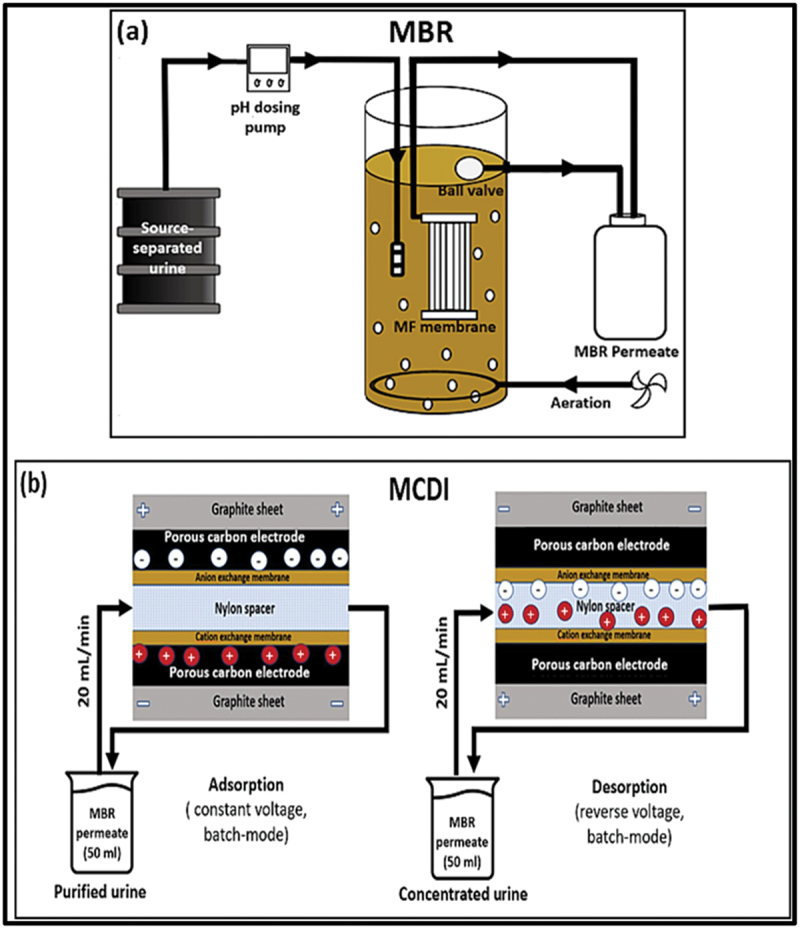
Schematic process of MBR and MCDI set up and operation [47], reproduced with permission obtained via copyright clearance center.
- *Gas Separation*: While CDI is mainly used for liquid-phase applications, recent advancement in supercapacitor-based CO_2_ capture [48] highlights the potential for applying similar principles of electrosorption to gas separation. Supercapacitors, particularly electric double-layer capacitors (EDLCs), utilize non-Faradaic processes similar to CDI, where ions are electrostatically adsorbed onto the electrode surface. This similarity suggests that advances in carbon-based materials and electrode design for supercapacitors could inspire innovations in CDI systems for gas-phase separations, such as CO₂ capture.
- *Resource Recovery*: CDI can be employed to recover valuable ions from various sources, such as extracting lithium from brine or mining runoff, leveraging its selective adsorption capabilities.
- *Chemical Processing*: CDI can assist in selective ion separation in chemical processes, aiding in purification or concentration of specific compounds.

CDI is primarily used for desalination of brackish water, treatment of industrial effluents, and for applications requiring low-salinity water such as agricultural irrigation. In addition to these traditional uses, CDI technology finds diverse applications across several innovative fields:

- *Electrochemical Energy Storage*: CDI technology is similar to electrochemical capacitors or supercapacitors. Some research explores its potential for energy storage by leveraging its ion-adsorbing properties
- *Environmental Monitoring*: CDI can be utilized to detect and remove trace levels of heavy metals and other contaminants from environmental water samples, serving as a monitoring tool for water quality assessments. Research on nutrient recovery from source-separated urine using a hybrid system of membrane bioreactor (MBR) and membrane capacitive deionization (MCDI) achieved significant removal and recovery rates for nitrates, phosphates, and ammonium, demonstrating energy efficiency across various operational voltages shown in Figure 3 [47].

Xing et al. [7] emphasize the versatility of CDI technologies, highlighting its use in water disinfection, its synergy with renewable energy sources for enhanced water treatment and resource recovery, and even in carbon dioxide capture processes. This broad array of applications underscores CDI’s adaptability and effectiveness in contributing to sustainable environmental practices. Such innovative applications are instrumental in pushing the boundaries of CDI technology, demonstrating its potential in addressing global water and energy challenges.

#### Applications for water purification

##### Ions

CDI is effective in removing various ions, including sodium, chloride, and heavy metals. In addition to removing common ions, CDI technology efficiently extracts rare earth elements (REEs) from geothermal brine using a solar-powered system. This method simultaneously purifies water and recovers valuable minerals, showcasing the broad applicability of CDI [49].

**Figure 4.**
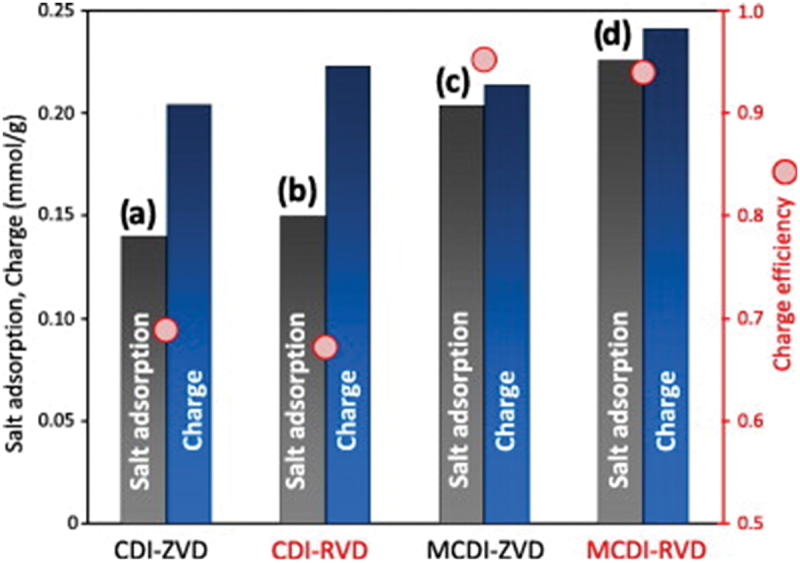
Salt adsorption and charge per cycle, and charge efficiency (total cycle duration 600 s) for (a) CDI-CV-ZVD, b) CDI-CV-RVD, (c) MCDI-CV-ZVD, and (d) MCDI-CV-RVD. Vcell = ±1.2 V, c_salt,in_ = 20 mM. (constant voltage (CV), zero-voltage desorption (ZVD), reversed-voltage desorption (RVD)) [11]. Reproduced with permission obtained via copyright clearance center.

Figure 4 presents a comparison of four CDI/MCDI configurations, highlighting differences in salt adsorption, charge storage, and charge efficiency. It shows that while traditional CDI setups zero-voltage (ZVD) and reversed-voltage (RVD) are capable of removing salt, the incorporation of membranes in MCDI systems significantly improves both desalination performance and charge utilization [11]. Notably, the MCDI-RVD setup achieves the highest salt adsorption and charge efficiency, indicating that combining membrane technology with reversed-voltage desorption enhances system performance and energy efficiency.

##### Organic

CDI can remove certain organic compounds, but its efficiency depends on the molecular size, the charge of the compounds and the membranes material properties. In an innovative approach, different porous membrane materials (polyethersulfone, polyamide, modified polyacrylonitrile) were duplex metal coated and electro-adsorption and electro-desorption of natural organic matter (NOM) were investigated and modelled [50,51]. In particular, larger organic molecules such as humic and fulvic acids can be removed by this CDI-based treatment approach. In addition to electrosorption and desorption, aqueous solutions are filtered by micro- or ultrafiltration membranes, resulting in low energy particle and partial NOM removal.

### Assessing CDI: advantages, disadvantages, and comparative analysis

CDI is often compared to reverse osmosis (RO), electrodialysis, and ion exchange systems. This section evaluates the performance of the comparative strengths and weaknesses of CDI, highlighting its optimal operational window and recent innovations.

#### Advantages of CDI

CDI offers unique benefits in specific water treatment scenarios:

- Energy Efficiency: CDI is more energy-efficient for low salinity water treatment, lacking the need for high-pressure pumps or heat sources, which are typically required in other desalination technologies like RO. This makes CDI particularly effective for applications such as brackish water desalination where lower energy and equipment costs are a priority [43]. Additionally, CDI’s ability to recover energy during the electrode discharging phase further enhances its overall energy efficiency [9].
- Operational Simplicity: The simplicity of CDI systems allows for ease of operation and minimal use of chemicals, enhancing its usability and reducing maintenance requirements [43].

- Regeneration Efficiency: CDI systems demonstrate ease of regeneration, which is crucial for continuous operation without the need for extensive downtime [43].

- Cost-Effectiveness: Recent studies highlight CDI as a cost-effective alternative to other desalination technologies, such as reverse osmosis (RO). The levelized cost of water (LCOW) refers to the total cost of producing one unit of desalinated water, taking into account capital, operational, and maintenance expenses over the system’s lifespan. CDI systems typically have a significantly lower LCOW, especially for water with low to moderate ion concentrations. This is due to CDI’s lower operational and maintenance costs, as well as its reduced energy consumption, making it an economical choice for desalination, particularly in applications where feed water has low to moderate salinity levels [9].

#### Disadvantages of CDI

Despite its advantages, CDI faces several challenges that may limit its applicability in certain scenarios:

- Sensitivity to High Salinity and Organic Loads: CDI is less effective in the presence of high salinity (more than 2 g/L TDS) or high concentrations of organic compounds [52]. At high ionic concentrations, electrode saturation and diminished electric double layer formation reduce performance and energy efficiency. These are not fundamental flaws, but rather design-specific characteristics that destine CDI’s optimal niche in low to moderate salinity conditions. For higher TDS levels, pressure-driven technologies like BWRO and SWRO demonstrate superior performance [42,53,54], as high salinity challenges the ion exchange capacities of CDI systems and can accelerate electrode degradation, thereby reducing operational lifespan and overall efficiency [52]. Additionally, organic compounds can foul CDI electrodes by forming scales or blocking pores, reducing electro-adsorption efficiency [19]. Hybrid systems such as CDI-electrooxidation (CDI-EO), ultrafiltration-CDI (UCDI), and CDI-electrochemical advanced oxidation processes (CDI-EAOP) have been proposed to overcome this limitation. While promising, these systems remain mostly at lab scale and require further field validation [55,56].
- Material and Operational Limitations: The effectiveness of CDI is heavily dependent on the properties of the electrode materials. The operational simplicity and energy efficiency can also be a drawback, as most existing CDI systems achieve limited energy efficiency from a thermodynamic perspective, often due to energy losses during the desalination process [57]. The cost of materials, particularly the electrode materials, is a significant challenge for the widespread adoption of CDI. The use of advanced carbon materials, while enhancing performance, increases the overall cost, making it less competitive for high-scale operations unless further material optimization and cost reduction are achieved [9].
- Inability to Remove Non-Ionic Impurities: While CDI can be effective in removing charged species, including microorganisms that often carry a charge in neutral water, it struggles to eliminate non-charge impurities such as parts of dissolved organic compound (DOC) and silicates [43]. This elimination can be significant in applications where the removal of non-ionic contaminants is required

Many of these limitations were already emphasized in Oren’s seminal 2008 review, which criticized CDI for low charge efficiency, limited salt removal capacity, and vulnerability to fouling and energy losses [14]. Although newer developments in electrode materials, membrane integration, and process configurations have addressed some of these issues, Oren’s critiques remain relevant as they continue to inform ongoing efforts to improve up-scaling and reliability.

In summary, CDI is conceptually similar to electrodialysis, but the later employs ion exchange membranes in a stacked electrode setup [58]. CDI, by contrast, is more suitable for waters with lower ionic strengths. Moreover, CDI offers several advantages over conventional pressure-driven systems and thermal technologies, as it does not require high-pressure pumps or heat sources, this makes CDI particularly effective for applications like brackish water desalination, where lower energy consumption and equipment are desirable.

#### Comparative context against RO and electrolysis

While RO is effective for high salinity waters, CDI requires less specific energy demand associated with RO systems, making it more suitable for lower salinity waters. Unlike electrodialysis, which uses ion exchange membranes and can handle a broader range of salinities, CDI is more restricted but offers simpler operations and lower energy costs.

Furthermore, the recent advances in hybrid CDI technologies such as RO-CDI [59], NF-CDI [60] and RO-MCDI-RED [61] show promise in addressing some of the inherent limitations of CDI, particularly in terms of scalability and operational efficiency [9]. Maheshwari et al. demonstrated that combining nanofiltration and CDI can effectively treat RO retentate while maintaining low energy consumption (~3.5 kWh/m^3^), making it suitable for high-performance wastewater treatment [60].

In a recent study, Lim et al. [62] quantitatively compared the performance of flow-electrode capacitive deionization (FCDI) with brackish water reverse osmosis (BWRO) and seawater reverse osmosis (SWRO). Their validated simulation model demonstrated that under low salinity conditions, FCDI could achieve a specific energy consumption (SEC) as low as 2.7% of BWRO’s SEC. Moreover, the study showed that with appropriate membrane area scaling and system optimization, FCDI can remain competitive even in higher salinity applications, offering a viable low-energy alternative to conventional membrane-based desalination technologies.

## Methods to study CDI

### Experimental

The current experimental research on CDI for water desalination is broadly focused on understanding and improving the technology through two key areas: electrode materials and reactor designs.

The key goals of this experimental research include:

#### Enhancing ion removal efficiency

The primary focus of experimental work is to increase the efficiency of ion removal, which directly correlates to the performance of the CDI system. This often involves improving the electrosorption properties of electrode materials or optimizing reactor configurations to enhance ion transport. Innovations such as those seen in Membrane Capacitive Deionization (MCDI) have shown how incorporating ion-exchange membranes in conjunction with porous electrodes can prevent co-ions expulsion from the electrodes, thus significantly enhancing ion removal efficiency [63]. This setup allows for more complete ion release with a reversed voltage, optimizing the efficiency of the deionization cycles.

#### Reducing energy consumption

Since CDI operates by applying a voltage across electrodes, minimizing energy consumption while maximizing desalination efficiency is crucial. Energy consumption in CDI can be significantly minimized by optimizing internal and external factors which has been reported by researchers where they experiment with different materials and reactor designs to achieve this balance which have been gathered in several review papers [64–66]. Jiang and his group in review paper [64] highlight that for example incorporating ion-exchange, employing flow-by configurations, and utilizing constant current charging modes are among the most effective strategies for reducing energy use. Furthermore, advancements in electrode materials, such as those with high wettability or semi-selective surface, contribute to more efficient ion adsorption and lower energy demand.

#### Improving selectivity

Selective ion removal in CDI is a critical area of research. Experimental efforts are aimed at designing materials or/and configurations that can target and remove harmful or unwanted ions from water while retaining beneficial ions. Recent studies, such as those by Tauk et al. highlight the advancements made in achieving better ion selectivity through innovative electrode materials [46,67].

#### Enhancing durability and longevity

The longevity and durability of CDI systems are important for practical applications. Experimental work often focuses on developing robust electrode materials and reactors that can withstand the wear and tear of continuous use, including addressing issues like fouling and electrode degradation [68]. The use of ion-exchange membranes in MCDI not only improves the system’s efficiency but also contributes to its durability by reducing the operational stress on the electrodes, thereby potentially reducing fouling and degradation [63]. This enhances the longevity and reliability of CDI systems under continuous operation.

As highlighted, the experimental research on CDI is typically centered around two main aspects of electrode materials and reactor design. Both of these areas are crucial because they directly impact the efficiency, effectiveness, and practicality of CDI for water desalination and other applications.

#### Electrode (material and synthesis methods)

Common materials include activated carbon, carbon aerogels, and carbon nanotubes. These materials are synthesized through various methods, including chemical vapor deposition, thermal treatment, and chemical activation.

Electrode materials are a crucial component in CDI, determining how effectively ions are adsorbed and removed from water. The current experimental focus on electrodes includes:

##### Material selection for electrode

The choice of electrode material is pivotal in determining the overall performance and efficiency of Capacitive Deionization (CDI) systems. Carbon-based materials, such as activated carbon, carbon nanotubes, and graphene, are the most commonly used due to their high surface area, excellent electrical conductivity, and ease of modification. These properties are essential for facilitating the migration of salt ions into the electrical double layers at the electrode/electrolyte interface when a voltage is applied [43].

Activated carbon and carbon cloths are traditionally favoured for their large surface areas, which provide extensive sites for ion adsorption. However, advancements in material science have led to the development of more sophisticated carbon materials. For example, ordered mesoporous carbons offer controlled ion transport, while carbon aerogels, with their high porosity, provide superior kinetics. Carbide-derived carbons allow for tuneable pore sizes, enabling specific ion targeting, and carbon nanotubes and graphene stand out for their exceptional electrical properties and large surface areas, significantly enhancing desalination capacities [11].

**Figure 5.**
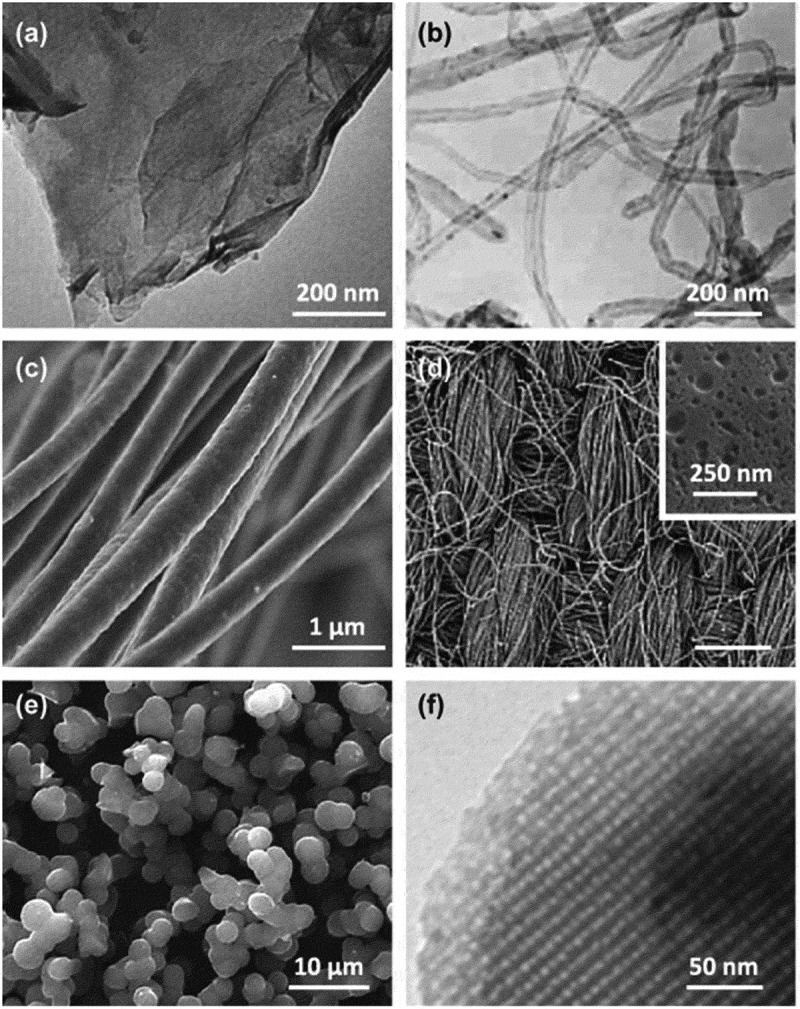
Selection of carbon materials used for CDI [11]. Graphene-like flake(a), muti-walled carbon nanotubes (b), electrospun fibers (c), activated carbon (d), carbon aerogel (e), and ordered mesoporous carbon. Reproduced with permission obtained via Copyright Clearance Center.

Figure 5 provides a selection of various carbon materials used for CDI applications, illustrating the diverse options available for optimizing performance in different operational contexts [11].

Recent studies have also focused on optimizing the electrochemical properties and surface characteristics of CDI electrodes by incorporating novel materials and advanced synthesis techniques. These include the use of hybrid materials, such as carbon-metal oxide composites, which have shown significant potential in improving ion adsorption capacity and overall system efficiency [9].

Graphene-based materials, in particular, have emerged as promising candidates for CDI applications due to their tuneable properties and unique structures [43]. These materials can be chemically and physically modified to optimize their intrinsic features and tailored into various structures, such as three-dimensional graphene foam with hierarchical porous structures, graphene/carbon composites, and nanoporous graphene membranes. These innovations not only enhance ion adsorption capacity but also improve the overall electrochemical performance and durability of the CDI system [69].

Beisch and Fiedler and Marx et al. introduced innovative carbon-based materials with properties that align well with the needs of CDI systems, despite not explicitly targeting CDI in their work [70,71]. One highlighted nanocarbon aerogels, such as Aerographite and globugraphite, which exhibit high porosity, lightweight structures, and exceptional electrical conductivity, qualities critical for enhancing ion transport and adsorption in CDI electrodes. These materials also offer scalability and tunable surface properties, suggesting significant potential for improving desalination efficiency and operational sustainability. Similarly, Marx et al. [71] proposed scalable methods for synthesizing hierarchical carbon foams with customizable porosity and multifunctional properties. The interconnected porous networks and adjustable surface chemistry of these foams facilitate efficient ion accessibility and reduce diffusion resistance, making them promising candidates for CDI applications. Together, these studies underscore the potential of adapting scalable and template-assisted synthesis techniques to develop cost-effective, high-performance materials tailored for CDI systems.

Laurent Joly and colleagues have highlighted the critical role of interfacial water dynamics and substrate-induced friction in confined systems, offering valuable guidance for CDI material optimization [72]. Their work emphasizes tailoring electrode surfaces, such as graphene, to control slip lengths and enhance ion transport efficiency at solid-liquid interfaces. These findings suggest potential improvements in ion accessibility and adsorption kinetics, key to advancing CDI electrode performance.

Earlier studies have highlighted the role of multi-walled carbon nanotubes (MWCNTs) in improving electrode performance in CDI systems. MWCNTs, due to their high aspect ratio and excellent electrical conductivity, can form highly efficient conductive networks within composite electrodes, enabling enhanced ion transport and adsorption. However, functionalizing MWCNTs to improve dispersion can introduce structural defects, which may influence the mechanical stability of the electrode [73]. This balance between enhanced conductivity and potential drawbacks underscores the need for optimized synthesis strategies, making MWCNTs a promising component in advanced CDI electrode design.

Recent advancements also include the development of hybrid materials, such as carbon-metal oxide composites and carbon-polymer composites. These materials leverage the inherent properties of carbon while incorporating metal oxides or polymers to boost specific capacitance and surface characteristics, leading to improved desalination performance. For instance, manganese oxide-coated carbon nanotubes have shown significant increases in specific capacitance and ion adsorption capacity, demonstrating the potential of these hybrid materials in advancing CDI technology [11].

Material selection in CDI is guided by critical factors such as salt removal efficiency, operational longevity, and cost-effectiveness. The continuous evolution of electrode materials, driven by ongoing innovations, aims to push the boundaries of what can be achieved in terms of energy efficiency, ion exchange capacity, and operational sustainability in CDI systems.

Tauk and colleagues [46] has a comprehensive review paper where they presented a *comprehensive review on electrode material* which provides an in-depth analysis of the latest finding with a strong focus on the evolution and optimization of electrode materials. Table 1 is a summary of the electrode materials used in Capacitive Deionization (CDI) based on their paper.

**Table 1.** Summary of electrode materials used in capacitive deionization (CDI).

Merial Type	Examples	Properties/Advantages	Limitations	References
Carbon-Based Electrodes				
Activated Carbon (AC)	–	High surface area, low cost	Low capacitance, poor electrical conductivity	[74,75]
Carbon Aerogels (CA)	–	High conductivity, high specific surface area	High cost, complex production	[10]
Mesoporous Carbon (MC)	–	Tuneable pore sizes, high surface area	Costly synthesis methods	[76]
Carbon Nanotubes (CNTs)	–	Excellent mechanical, electrical, and thermal properties	Expensive, challenging to process, toxicologically problematic, impurities after production	[77]
Graphene	Graphene composites (e.g. with AC)	High surface area, superior conductivity, mechanical strength	Expensive	[78]
Carbon-Transition Metal Oxide Composites	TiO_2_, ZnO composites	Improved electrochemical properties, enhanced ion adsorption capacity	Complex synthesis, high cost	[79,80]
Faradaic Electrodes				
Faradaic Materials	–	High ion storage capacity through redox reactions	Potentially complex manufacturing	[81,82]
Composite Materials				
Carbon-Metal Oxide Composites	Carbon-TiO_2_, Carbon-ZnO	Enhanced wettability, improved ion adsorption, better electrochemical performance	High material costs, complex synthesis	[83]
Hybrid Materials	Carbon-Faradaic combinations	Combines high surface area and redox activity, improved desalination capacity	More complex to design and optimize	[36,84]

##### Material modification

Achieving high-efficiency CDI performance relies heavily on the rational design and modification of electrode materials. To systematically understand how modifications influence performance, we categorize approaches based on key material properties: (i) surface area and porosity, (ii) electrical conductivity, (iii) surface functionality, and (iv) electrochemical stability.

###### Surface area and porosity

**Figure 6.**
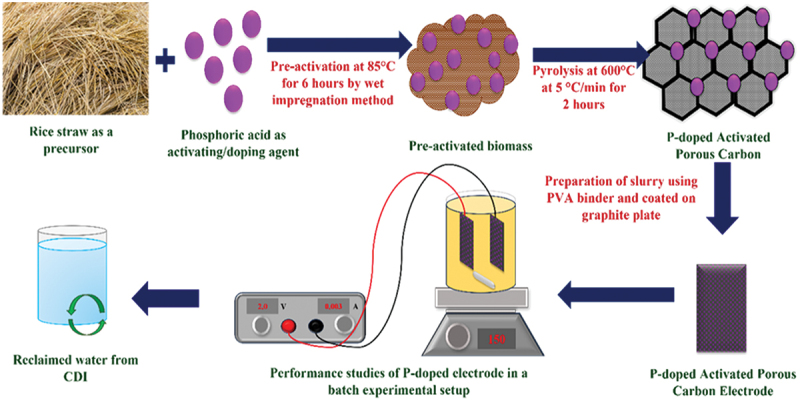
Overview of the P-doped bio carbon electrode for Cr(VI) reclamation [87] reproduced with permission obtained via copyright clearance center.

Enhanced ion adsorption depends largely on the specific surface area and pore structure of the electrode. Hierarchical porous structures improve ion transport and increase available adsorption sites. For instance, carbon materials derived from biomass (e.g. rice straw) offer naturally porous frameworks. Ganesan et al. [85] developed phosphorus-doped carbon electrodes from rice straw, which demonstrated high surface area and improved Cr (VI) ion removal due to increased pore accessibility and charge storage (Figure 6).

High conductivity ensures efficient electron transport during charging/discharging. Doping carbon materials with heteroatoms like nitrogen or introducing conductive metal oxides helps achieve this. For example, Cheng et al. [86], reported that nitrogen doping improves charge transfer and capacitive performance. Metal oxide-carbon composites can further boost conductivity and capacitance through synergistic effects. In line with this [87], showed that heteroatom doping not only improves the electrical conductivity by introducing charge carriers and structural defects but also enhances the electrochemical performance of carbon materials through improved ion mobility and charge distribution. These principles, while explored for supercapacitors, are directly translatable to CDI electrodes, particularly in applications requiring high-rate performance and low-resistance pathways. Additionally, they demonstrates that interfacial engineering of 2D heterostructures such as WO₃/WSe₂ nanosheets can significantly improve electron transport and Na^+^ ion diffusion by creating conductive pathways and optimized charge distribution across interfaces [88]. While their study centers on sodium-ion storage, these strategies are highly relevant to CDI, where enhancing interfacial conductivity and ion kinetics remains a key challenge.

###### Surface functionality and charge selectivity

Functional groups on the electrode surface influence ion selectivity and resistance to fouling. AlMarzooqi et al. highlighted that introducing oxygen- or nitrogen-containing groups can promote specific ion adsorption. Metal oxide additions can also improve selectivity towards multivalent ions due to their chemical affinity [6]. According to Shinde et al. [87] heteroatoms such as nitrogen, sulfur, and phosphorus increase the surface polarity and create additional active sites, improving wettability and enabling stronger interactions with ionic species. Furthermore, multi-doping strategies offer the possibility of synergistic effects that fine-tune the surface chemistry for enhanced ion selectivity, though their complex behavior still requires deeper understanding.

In a similar context, layered double hydroxides (LDHs), such as La-based LDH on activated carbon or CoNi – LDH composites, have shown excellent potential for selective ion removal in CDI. LDHs possess abundant surface functional groups and tuneable interlayer spaces, making them suitable for capturing specific anions (e.g. Cl^−^, PO₄^3−^) via intercalation mechanisms. Recent studies have demonstrated that LDH-modified carbon electrodes enhance wettability and charge-transfer kinetics while improving ion selectivity and cycling stability [89,90].

###### Electrochemical stability and faradaic behavior

**Figure 7.**
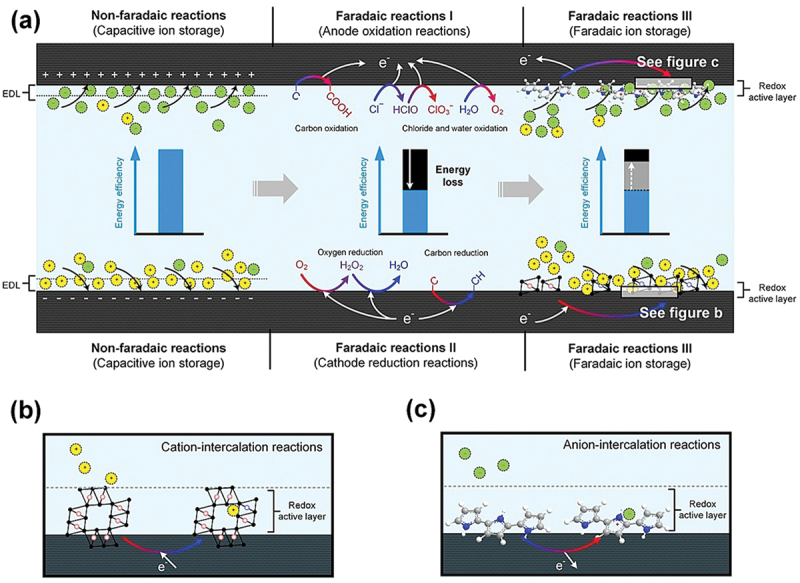
Schematic presentation of three types of Faradaic processes (anodic oxidation, cathodic reduction and Faradaic ion storage processes) [69]. Reproduced with permission obtained via copyright clearance center.

Material stability under operational voltage is vital for long-term CDI performance. Faradaic reactions (e.g. redox activity or unwanted by-product formation) can degrade electrode integrity. Zhang et al. [69] illustrated how material selection and operational mode influence anodic oxidation, cathodic reduction, and Faradaic ion storage (Figure 7). Engineering materials with stable redox properties or suppressing microbial degradation reactions through coatings and doping can prolong electrode lifespan.

In this context, transition metal oxides such as tungsten oxide (WO₃) offer promising pathways to balance Faradaic activity and stability. As reported by Shinde et al., WO₃-based electrodes exhibit excellent pseudocapacitive behavior, structural tunability, and redox stability, which have already shown success in supercapacitors and batteries [91,92]. The integration of such materials into CDI electrodes especially as hybrids with carbon can help enhance ion storage while minimizing degradation under prolonged cycling. Additionally, these composites can improve structural robustness and extend the operational potential window, which are crucial for maintaining electrochemical integrity in CDI applications.

Similarly, LDH-based composites, such as hollow CoNi – LDH structures grown on carbon substrates, provide reversible anion intercalation and stable Faradaic behavior, combining the benefits of capacitive and pseudocapacitive mechanisms. These systems have demonstrated improved salt adsorption capacity, long-term cycling performance, and suppression of parasitic reactions during CDI operation [93]. Their structural stability and tunable redox properties offer a valuable direction for durable, high-efficiency CDI electrode design.

Incorporating these design principles, rather than applying unsystematic material modifications, enables a rational and predictive framework for CDI electrode optimization. This strategy clarifies the relationship between between electrode structure, composition, and surface chemistry and key performance metrics such as capacitance, ion adsorption rate, and cycling stability.

In summary, systematically tailoring key material properties, including surface area and porosity, conductivity, functional chemistry, and electrochemical stability enables a rational, predictive approach to CDI electrode design. This structured strategy helps establish clear structure – property – performance relationships, moving beyond empirical trial-and-error modifications and toward optimized electrodes with improved adsorption capacity, selectivity, and durability.

###### Synthesis methods

The synthesis process of electrode materials significantly influences their properties, including porosity, surface area, and electrochemical performance. Common synthesis methods for CDI electrodes include:

- Chemical Vapor Deposition (CVD): A popular technique for synthesizing high-purity carbon materials like carbon nanotubes and graphene. This method involves the deposition of gaseous reactants onto a substrate to form a thin film, providing control over the material’s structure and properties.
- Pyrolysis: A thermal decomposition process where organic precursors are heated in the absence of oxygen to produce carbon materials like activated carbon and carbon aerogels. This method is known for producing materials with high surface area and controlled pore size.
- Hydrothermal Synthesis: This method involves the crystallization of substances from high-temperature aqueous solutions at high vapor pressures. It’s particularly useful for incorporating metal oxides into carbon matrices to create hybrid materials with enhanced electrochemical properties.

The choice of synthesis method is crucial, as it directly impacts the efficiency of the CDI process by affecting the electrode material’s characteristics, such as porosity, surface area, and ion adsorption capacity. Advanced synthesis techniques are continually being developed to optimize these properties for better performance in CDI applications.

##### Electrode architecture

Electrode architecture refers to the physical design and structural arrangement of electrode materials within a CDI system. This includes the internal structure of the electrode, such as pore size and distribution, as well as the overall configuration and placement of electrodes within the CDI cell. The architecture of the electrodes is crucial in determining the efficiency of ion transport, adsorption capacity, and overall desalination performance of the CDI system.

Key Aspects of Electrode Architecture:

1. Pore structure
  - Micro-, Meso-, and Macropores: The electrode’s pore structure plays a significant role in ion transport and adsorption. Micropores offer a high surface area for ion adsorption, mesopores facilitate faster ion diffusion, and macropores enhance fluid flow. A well-balanced pore structure, often referred to as a hierarchical pore structure, can significantly improve the efficiency of CDI by providing multiple pathways for ions to access the adsorption sites.
  - Optimizing Pore Size: Recent studies, such as those by Breitsprecher et al. (2018), have demonstrated that carefully controlled pore dimensions in nanoporous electrodes can improve ion movement by minimizing co-ion trapping, which occurs during rapid charging [94]. By selecting optimal pore sizes, CDI systems can enhance charging efficiency, ultimately contributing to faster ion adsorption and desorption.
2. Geometry
  - Electrode Shape and Configuration: The geometry of the electrodes, including their shape and spatial arrangement within the CDI cell, can significantly influence performance. Common geometries include flat plates, 3D structures, and flow-through designs. Innovations in electrode geometry, such as using 3D-printed structures or patterned surfaces, aim to increase the effective surface area and optimize the flow of water through the cell, thereby enhancing ion removal efficiency [6].
3. Configuration
  - Electrode Placement in CDI Systems: The way electrodes are configured within the CDI cell impacts the flow dynamics and efficiency of ion removal. Typical configurations include:
    - Flow-by: Where water flows parallel to the electrode surface.
    - Flow-through: Where water flows through the electrode material itself, ensuring more direct contact between the ions and the electrode surface.
    - Hybrid Configurations: Combining different electrode placements or integrating other functional materials (e.g. ion-exchange membranes) to improve performance and selectivity.

Recent developments in electrode architecture have focused on creating hierarchical structures that incorporate multiple pore sizes and optimizing the spatial arrangement of electrodes within the CDI cell. For example, 3D-printed electrodes with precise pore structures have been developed to maximize the ion transport rate while minimizing energy consumption. Moreover, integrating advanced materials like graphene into the electrode architecture has shown promising results in increasing desalination efficiency and reducing fouling

#### Reactor design/cell architecture

CDI reactors are commonly designed with either a flow-through or flow-by configuration, each of which significantly impacts ion removal efficiency. The design of the reactor dictates how water flows through the system and interacts with the electrodes, influencing ion transport and adsorption capacity. Experimental efforts in reactor design focus on optimizing water flow dynamics and maximizing contact with the electrode surfaces to enhance desalination performance. Key areas of focus include:

##### Reactor configuration

**Figure 8.**
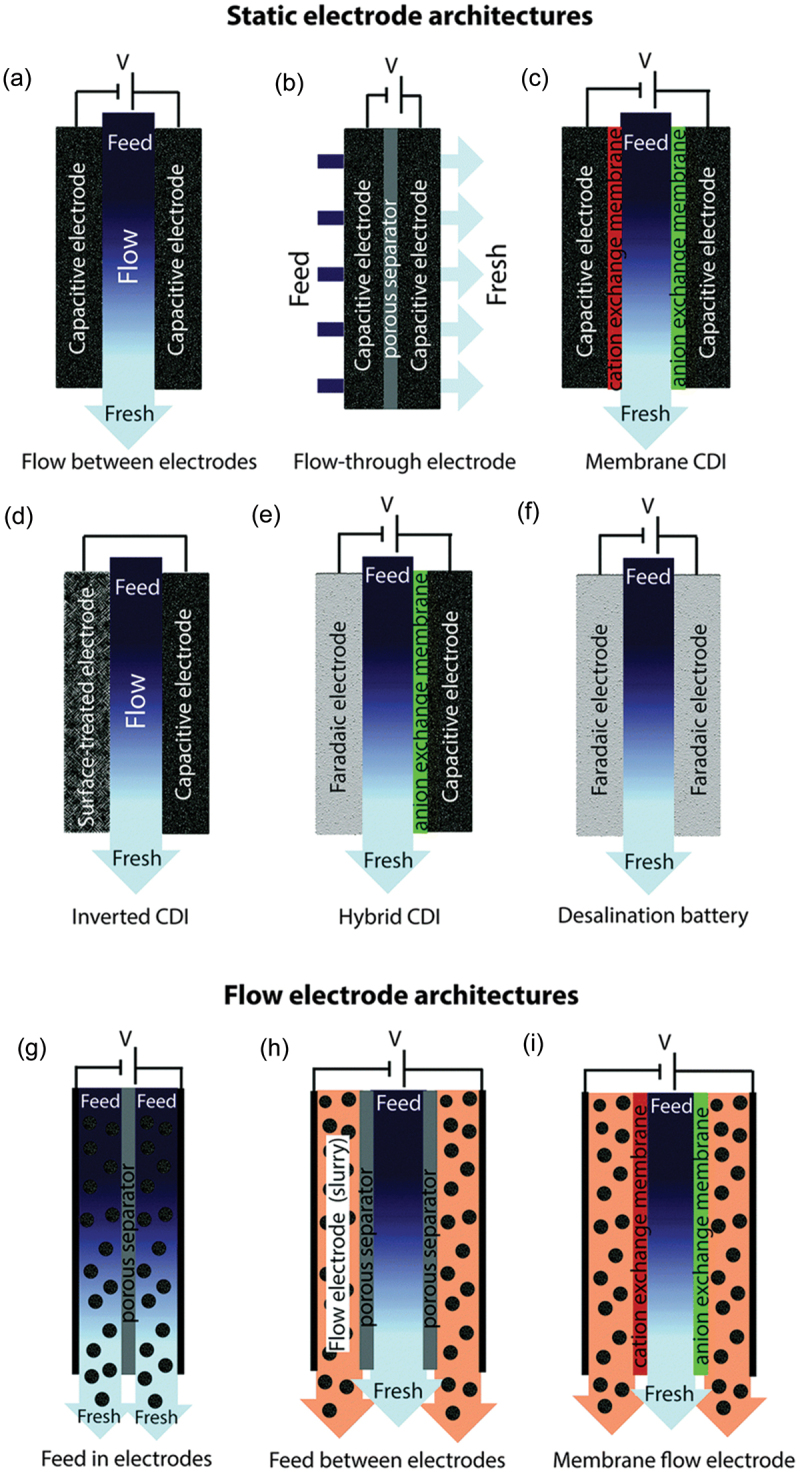
(a)–(d) CDI architectures using static electrodes, including: (a) flow-between electrodes, (b) flow-through electrode, (c) membrane CDI, and (d) inverted CDI. (e) and (f) show architectures which utilize static electrodes that depart from purely capacitive behaviour, including (e) hybrid CDI, and (f) a desalination battery. (g)–(i) show CDI architectures with flow electrodes, including systems with (g) feed-in electrodes, (h) feed-between electrodes, and (i) membrane flow electrode CDI [3]. Reproduced with permission obtained via copyright clearance center.

The design of CDI reactors, including flow-through and flow-by configurations, impacts ion transport and removal efficiency. Many review papers have gathered and analysed the results of experimental studies which explore how these configurations affect performance under different operating conditions [3,95]. The configuration of the entire capacitive deionization (CDI) system, or cell architecture, plays a crucial role in the technology’s overall performance and efficiency. These cell architectures aim to optimize the operational efficiency of CDI systems by enhancing ion removal capabilities and reducing energy consumption. Figure 8 illustrates various configurations of capacitive deionization (CDI) cells, including traditional flow-by and advanced membrane CDI (MCDI) systems, highlighting innovations like ion exchange membranes to improve ion removal efficiency and extend electrode longevity [3]. These configurations demonstrate the evolution of CDI technology from basic setups to complex systems incorporating additional features for enhanced performance and durability [95].

Hybrid systems, for example, utilize both capacitive and Faradaic processes to increase charge storage capacity and energy efficiency, representing a significant evolution in CDI technology. The integration of membrane technologies in MCDI systems helps improve selectivity and increase salt removal efficiency by effectively managing ion movement across the cell.

##### Flow dynamics

Optimizing flow dynamics within a CDI reactor is crucial for efficient ion removal, as it promotes even ion distribution and maximizes contact between water and electrode surfaces, which is essential for effective desalination. Research in this area emphasizes refining flow paths to minimize resistance and improve ion transport to electrode surfaces, resulting in higher desalination rates and overall system efficiency [96].

As explained by Qu et al. [97], in flow-through electrode CDI systems, ion transport is governed by two main regimes: the advection-limited regime and the dispersion-limited regime. In the advection-limited regime, which occurs at higher flow rates, ions are quickly carried to electrode surfaces, maximizing adsorption rates and ensuring uniform ion distribution. In contrast, at lower flow rates, the system operates within the dispersion-limited regime, where ion transport relies more on diffusion than flow. This can lead to concentration gradients, slowing salt removal and making electrode porosity optimization more significant to maintain consistent desalination performance.

##### Stack design

CDI reactors often use stacked electrode pairs to enhance performance by increasing the active surface area for ion adsorption. The arrangement and number of electrode pairs within a stack influence both the salt removal efficiency and the reactor’s energy requirements. Optimal stack designs balance electrode spacing and flow paths, minimizing pressure drop and maximizing ion transport to the electrodes. Studies suggest that symmetrical flow paths and short, consistent pathways can help prevent ‘dead zones’, ensuring a more uniform ion adsorption across the stack, which is especially crucial for scaling CDI systems for larger applications [98].

##### Operational parameters

Experiments often focus on the operational parameters of the reactor, such as voltage, flow rate, and cycle time. These parameters influence ion adsorption capacity, energy consumption, and electrode lifespan.

Martinez et al. in their research study successfully optimized a CDI cell for water desalination by evaluating the effects of electrode thickness and flow rate, achieving a specific absorption capacity of 10.2 mg/g and specific energy consumption of 217.8 Wh/m^3^ at a flow rate of 55 mL/min, thus providing valuable insights for improving CDI system efficiency [99]. Peng et al. demonstrated that chemically modifying graphene with strong acids significantly enhances its wettability, electrochemical properties, and electrosorption capacity, achieving a 3.9 times improvement in NaCl removal efficiency in CDI processes compared to unmodified graphene [100].

**Figure 9.**
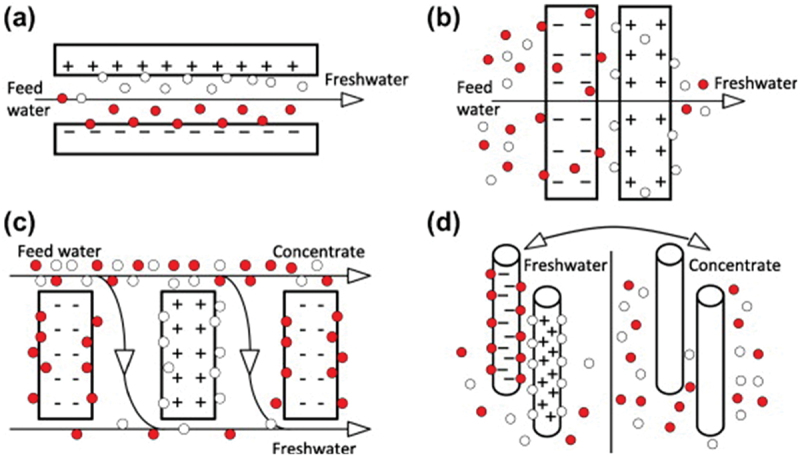
Overview of most relevant CDI system geometries. (a) Flow-by mode, (b) flow-through mode, (c) electrostatic ion pumping, and d) desalination with wires [11]. Reproduced with permission obtained via copyright clearance center.

Figure 9 is detailing different reactor configurations and their operational nuances, show how CDI systems can be structured for various applications.

These experimental developments in reactor design and operation form the foundational context for the theoretical and simulation approaches discussed in the next section, where modeling and predictive tools are used to deepen and generalize our understanding of CDI system behavior.

### Theory and simulation

While section 2.1 focuses on experimental aspects of reactor design and operation, this section complements it by exploring how simulation techniques predict behavior, optimize performance, and uncover underlying mechanisms in CDI systems. Simulation plays a crucial role in advancing CDI technology for water desalination by providing detailed insights into ion transport mechanisms, optimizing electrode architectures, and predicting system performance under different operating conditions. Simulations enable researchers to test various configurations and material properties, which can be time-consuming and costly to trial experimentally. This approach accelerates the development of more efficient CDI systems, helping to improve desalination performance, energy efficiency, and selectivity. The importance of simulation in this context stems from several key factors:

- Understanding Complex Phenomena: CDI involves intricate processes such as ion transport, electrochemical processes, and fluid dynamics. Simulations enable researchers to study these phenomena in detail, providing insights that are difficult or impossible to obtain through experimental work alone.
- Design Optimization: Simulations allow for the optimization of both electrode materials and reactor designs before actual fabrication. This helps in identifying the most promising configurations and materials, saving time and resources in the development process.
- Cost and Time Efficiency: Conducting physical experiments can be expensive and time-consuming, especially when testing multiple variables. Simulation provides a cost-effective way to test different scenarios and identify the most promising approaches for experimental validation.
- Predictive Modeling: Simulation plays a pivotal role in capacitive deionization (CDI), particularly for predicting the performance of CDI systems under varying operational conditions. These predictive models are crucial for transitioning from laboratory-scale experiments to full-scale real-world applications. The recent review by Gamaethiralalage et al. underscores the importance of advanced modeling techniques in enhancing ion selectivity within CDI systems [101]. These simulations focus on the selective separation and recovery of target ions, which is essential for resource recovery and environmental remediation applications. They detail the development of new theoretical models that accurately predict the interactions and dynamics of specific ions within various electrode and membrane configurations, thereby improving the design and efficiency of selective CDI processes. By incorporating these sophisticated modeling approaches, researchers can better understand the nuanced behaviors of CDI systems, leading to more effective and targeted applications in water treatment technologies. This enhanced understanding allows for the design of CDI systems that are not only more efficient but also tailored to specific purification needs, making the technology more adaptable and effective in practical applications. The dynamic Langmuir (DL) model, as demonstrated by Nordstrand et al., provides a simplified yet effective approach for modeling the performance of CDI systems, enabling predictions of ion adsorption and effluent concentration under various operational conditions [102]. These advancements in predictive modeling are instrumental in optimizing the ion selectivity of CDI, thereby broadening its applicability and efficiency in real-world scenarios.
- Guiding Experimental Research: Simulation studies often guide experimental research by identifying key parameters and conditions to test. This synergy between simulation and experimentation accelerates the development of more efficient and effective CDI systems.

Given these benefits, simulation is an invaluable tool in CDI research, complementing experimental work to enhance our understanding and improve the technology’s performance.

Beyond individual methods, a recent review by Zhang et al. offered an integrated outlook on cutting-edge numerical simulations in CDI, highlighting opportunities to improve model accuracy, reduce computational costs through machine learning, and incorporate life-cycle and techno-economic considerations for sustainable application [103]. These perspectives underline the need for multi-scale, interdisciplinary modeling paradigms to drive the next generation of CDI research.

#### Reactor modeling

Reactor modeling in CDI primarily aims to simulate and understand the behavior of ions within the reactor, focusing on ion transport and electrochemical reactions. Models like the Nernst-Planck (NP) equations are extensively used to describe ion transport dynamics in porous electrodes, capturing the effects of electric fields and concentration gradients on ion distribution. The NP equations describes how ions move under the influence of diffusion, migration due to electric fields, and convection. These models provide a framework to understand ion transport in CDI reactors, especially under varying operational conditions [96,104,105].

In CDI, reactor modeling using NP and PNP models focuses on understanding how factors like flow rates, voltage application, and electrode microstructure, which significantly impact the desalination performance. For instance, studies employing NP models analyze the influence of concentration gradients and flow rates on desalination efficiency [104]. Meanwhile, the PNP models capture the interplay between ion transport and electrostatic interaction within porous electrodes, offering deeper insights into optimizing CDI reactor designs.

In more complex systems where the electric field is influenced by the ions themselves, the Poisson-Nernst-Planck (PNP) model is applied. The PNP model extends the NP approach by coupling it with Poisson’s equation, which accounts for the electrostatic potential generated by the distribution of charges within the system. This self-consistent approach allows for a more accurate simulation of ion adsorption and transport, especially in the formation of EDLs at the electrode-electrolyte interface [106].

Recent advancements have further extended CDI reactor modeling into three dimensions. A notable contribution by Nordstrand et al. introduced the first fully coupled, spatiotemporal 3D model for electrochemical deionization, utilizing an electrolytic capacitor (ELC) framework to simulate CDI devices under realistic operational conditions. This model incorporates detailed representations of ion transport, leakage currents, and charge efficiency within asymmetric flow-through reactor architectures [107]. Implemented using finite element methods in COMSOL, it solves coupled fluid dynamics, ion flux, and electric field distributions across all device dimensions. Interestingly, the study demonstrated how flow asymmetries and dead zones can impact performance, showing that even with such imperfections, adequate desalination can be maintained under normal operating conditions. This modeling approach represents a significant step forward in reactor-level CDI simulations, providing tools to optimize not only electrode and cell design but also operational strategies in more realistic, three-dimensional configurations.

In parallel, Haverkort et al. (2024) developed an analytical reactor model for flow-by CDI under mass-transport-limited conditions, reducing the system to two coupled partial differential equations and solving them using the Lambert W function [108]. he model was validated against full 2D computational simulations and shown to provide accurate predictions under mass-transport-limited conditions. Furthermore, the authors used the analytical solutions to derive expressions for optimal design parameters such as electrode and spacer thickness, channel length, and operating pressure. Their analysis revealed that productivity can be significantly improved by up to an order of magnitude through geometric optimization, and they identified an optimal electrode-to-spacer thickness ratio of approximately 0.17. While particularly suitable for low-salinity regimes, the generic structure of the model makes it extendable to other domains such as phase-change batteries, thermal sorption systems, or electrochemical microreactors, making it a versatile tool for CDI system design and beyond.

Additionally, Lim et al. presented a numerical model tailored to flow-electrode CDI (FCDI), accounting for energy losses in various cell components such as membranes, electrodes, and electrolyte flow channels [62]. Their model was validated against pilot-scale experimental data and used to optimize energy efficiency and membrane area for desalination across different salinity levels.

By analyzing these factors through numerical simulations using NP or PNP models, reactor modeling helps optimize the design and operational parameters of CDI systems. This approach is crucial for enhancing in removal efficiency, reducing energy consumption, and improving overall system performance under different operational conditions [96,105].

#### Computational fluid dynamic (CFD)

CFD deals with the detailed analysis of fluid flow within the CDI reactor. It uses numerical methods and algorithms to solve and analyze fluid flow, turbulence, and heat transfer. CFD simulations help optimize reactor designs by examining how fluid interacts with the electrodes, ensuring uniform distribution, and minimizing dead zones or short-circuiting. CFD focuses more on the physical movement of water through the reactor and the associated impacts on ion transport.

Several studies have applied CFD to conventional CDI systems to optimize flow characteristics and improve ion removal efficiency. For example, a recent study using rice husk-derived activated carbon electrodes combined CFD modeling with experiments to evaluate flow patterns in a square CDI channel [109]. The simulations showed that increasing the feed flow rate reduced stagnant regions, leading to improved mass transport and higher electrosorption efficiency for hexavalent chromium. These results were validated experimentally, with RHWBAC electrodes achieving up to 85% removal from a 10 mg L^−1^ solution. The adsorption behavior followed Langmuir and Redlich – Peterson isotherms, while the kinetics matched a pseudo-first-order model. This case highlights the effectiveness of CFD in guiding operational improvements even without altering electrode material or reactor design.

Recent CFD studies also focus on scaling up Flow Capacitive Deionization (FCDI) modules. He et al. developed a 3D CFD model to assist in the design of an FCDI unit using tubular electrodes, which expand the effective membrane surface area available for salt removal [110]. The simulations identified that narrow gaps between adjacent tubular electrodes contributed most significantly to salt removal, owing to enhanced flow – electrode interaction in those regions. However, the model also highlighted that low flow velocities could limit this advantage. This work demonstrates how CFD can be used as a predictive tool for performance bottlenecks in FCDI design and optimization, especially during scale-up.

In Flow Capacitive Deionization (FCDI), fluid dynamics become significantly more complex due to the use of flow electrodes, which are non-Newtonian shear-thinning slurries composed of suspended carbon particles in water. Unlike conventional CDI systems where feedwater behaves as a Newtonian fluid with constant viscosity flow electrodes exhibit viscosity that decreases with increasing shear rate, making their flow behavior highly dependent on local shear conditions and channel geometry.

Saif et al. investigated this behavior through a combined CFD and experimental study, revealing how different channel geometries influence shear rate, viscosity, flowability, and mixing within FCDI systems [111]. Their results demonstrated the critical role of non-Newtonian fluid dynamics in channel design, especially to avoid clogging and enhance salt removal efficiency. For a more detailed discussion and visualization of these findings, refer to Section 2.3.

While Nordstrand et al. in their paper do not specifically address CFD, however they highlight the importance of fluid dynamics in CDI. Integrating CFD with the reactor models presented in the paper could further enhance the understanding and optimization of fluid flow in CDI systems, ultimately leading to more efficient designs [102].

Despite its strengths, CFD modeling requires high computational resources and accurate input data for boundary conditions, often necessitating experimental calibration. Nonetheless, it serves as a critical link between conceptual reactor design and real-world implementation, complementing electrochemical and molecular-level simulations. Its continued integration with experimental workflows is essential for advancing next-generation CDI systems.

#### Molecular dynamic simulation

MD simulation provide valuable insights into ion interactions at the molecular level, which is critical for designing advanced electrode materials in CDI systems. MD simulations involve studying the behavior of individual molecules and ions at the atomic level. They provide insights into how ions interact with electrode surfaces, how these interactions affect adsorption, and how molecular-level phenomena influence overall system performance. MD focuses on understanding the detailed interactions at the molecular level, which is crucial for designing advanced electrode materials with specific properties.

For instance, it has been demonstrated that through MD simulations that geometry of graphite electrodes, specifically the curvature of their surfaces, significantly influences the differential capacitance and ion distribution at the electrode-electrolyte interface [112]. Concave surfaces were found to enhance ion storage compared to convex ones due to modification in the EDL. This highlights the importance of electrode design in CDI systems, where optimizing the curvature and surface architecture can improve ion adsorption efficiency and overall desalination performance.

Recent studies emphasize the role of the water contact layer at solid-liquid interfaces, which directly influences hydration, transport properties, and ion dynamics [113]. The structuring of interfacial water, as governed by the substrate material, affects slip lengths and friction at the solid-liquid interface, impacting fluid dynamics in confined systems like CDI cells. Tailoring interfacial properties to optimize water structuring and ion mobility offers promising avenues for enhancing CDI efficiency by improving ion transport and adsorption mechanisms.

For a different material for example, MD simulations have highlighted the potential of graphene oxide (GO) and graphite oxide (GTO) for CDI applications, revealing their selective ion adsorption capabilities and enhanced water transport properties. The hydrophilic functional groups on GO surfaces, such as hydroxyl and epoxy groups, promote strong interactions with sodium ions (Na^+^), crucial for effective desalination [114]. The structural features of GO, including surface corrugations, enhance electrochemical performance by improving charge storage and current density, demonstrating the critical role of surface functionalization and structural optimization in CDI electrode design.

Research on interfacial hydrodynamics has further emphasized the critical role of fluid behavior in nanoscale systems. Herrero et al. revealed that supercooled water exhibits significantly increased slip at the solid-liquid interface due to enhanced molecular dynamics, reducing energy barriers for ion transport [115]. These findings are particularly relevant for CDI systems, as reduced interfacial friction directly correlates with improved ion mobility and lower energy consumption during desalination processes. Incorporating such insights into CDI electrode design could lead to materials that optimize interfacial hydrodynamics for enhanced system performance.

In addition, Oga et al. introduced a theoretical framework to model frequency-dependent liquid-solid friction at the atomic level [116]. This approach highlights the impact of electrode surface textures on minimizing frictional losses, which are critical for optimizing ion transport efficiency in CDI. By aligning molecular-scale fluid dynamics with practical material engineering, this work provides a pathway for integrating atomic-level insights into the development of advanced CDI electrode surfaces with tailored frictional properties.

**Figure 10.**
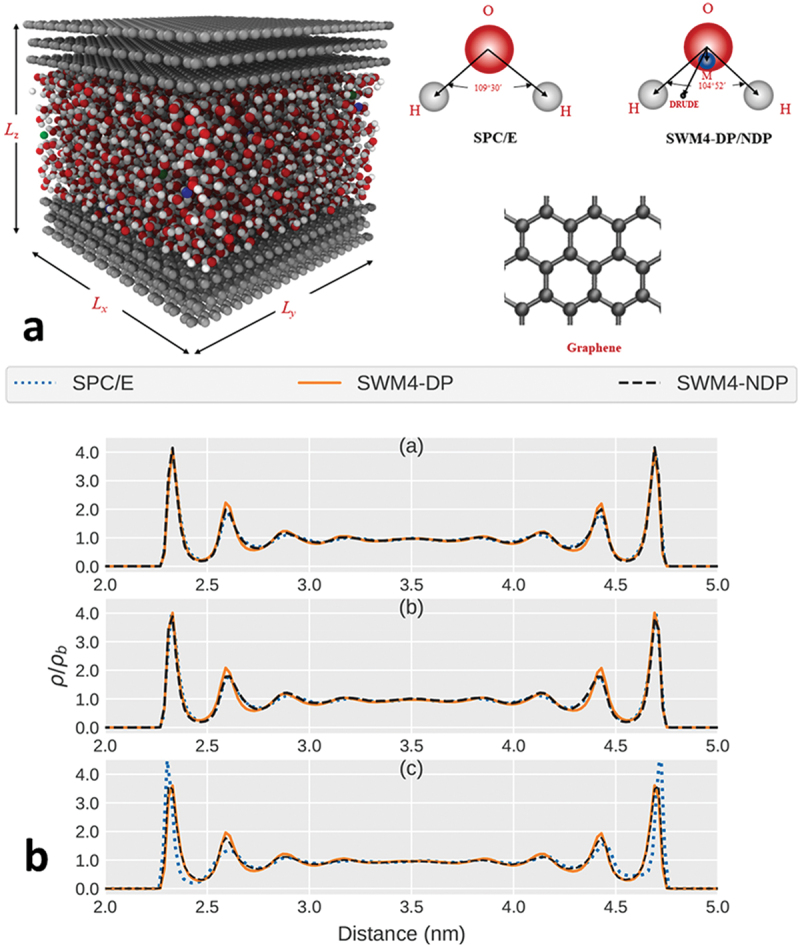
Molecular dynamics simulation of confined water in graphene slit pores [117]. (a) Simulation snapshot of the slit pore filled with water as well as sodium and chloride ions. The colors are as follows: gray is for C atoms of graphene sheets; blue is for Na atoms; green is for Cl atoms; red and white are for O and H atoms in water molecules, respectively. (b) Density profiles of water calculated using SPC/E, SWM4-DP, and SWM4-NDP force fields in the graphene slit pore at applied potentials of −1 V (a), 0 V (b), and +1 V (c). Densities are normalized by bulk water density. Reproduced with permission obtained via copyright clearance center.

Moreover, a recent study by [117] employed LAMMPS-based MD simulations to elucidate the role of dielectric effects and nanoscale water structuring in CDI systems. By comparing polarizable and nonpolarizable water force fields in graphene slit pore models, they found that polarizable models slightly increased the dielectric constant and enhanced the hydrogen bonding dynamics of confined water. These phenomena significantly influenced the Stern layer capacitance and electrosorption behavior, underscoring the importance of accurately capturing dielectric response and interfacial water structure in nanoscale CDI modeling. Figure 10 illustrates both the simulation setup and key findings: Figure 10a shows a snapshot of the graphene slit pore filled with water and ions, while Figure 10b presents normalized water density profiles across the pore under different applied electric potentials and water models, highlighting how polarizability and confinement influence interfacial structuring and performance.

MD simulations can complement Ordinary Differential Equations (ODEs) models by offering detailed insights into the interactions at the molecular level, which is crucial for designing advanced electrode materials with specific properties [118].

#### Numerical modeling

Numerical modeling involves solving the equations governing ion transport and electrical potential distribution, and provides insight into optimising CDI performance. The use of the DL model in MATLAB, as applied by Nordstrand et al., exemplifies how numerical modeling can streamline the prediction of CDI dynamics, offering a practical tool for adjusting experimental conditions to achieve desired desalination outcomes [102].

Numerical modeling in CDI encompasses a broader range of approaches in solving mathematical equations that describe ion transport, electrical potential distribution, and other phenomena within the reactor. These models may include solving differential equations or using finite element analysis to study various aspects of the CDI process. The primary focus is on obtaining accurate solutions to complex equations that describe the system’s behavior, thus providing insights for optimizing performance based on different parameters. Many researchers have contributed to advancing numerical modeling in CDI. For instance, Biesheuvel and Bazant [119] developed a mean-field theory for capacitive charging and desalination by porous electrodes, which delves into nonlinear dynamics. Ramachandran et al. further explored frequency analysis and resonant operation, adding depth to the understanding of CDI’s dynamic response [120]. Liu et al. presented a two-scale numerical model simulating ion transport and adsorption in porous electrodes, enhancing the understanding of desalination mechanisms [96]. Additionally, Saffarimiandoab et al. employed interpretable machine learning to analyze electrode and process features, showcasing an innovative approach to performance prediction [121].

**Figure 11.**
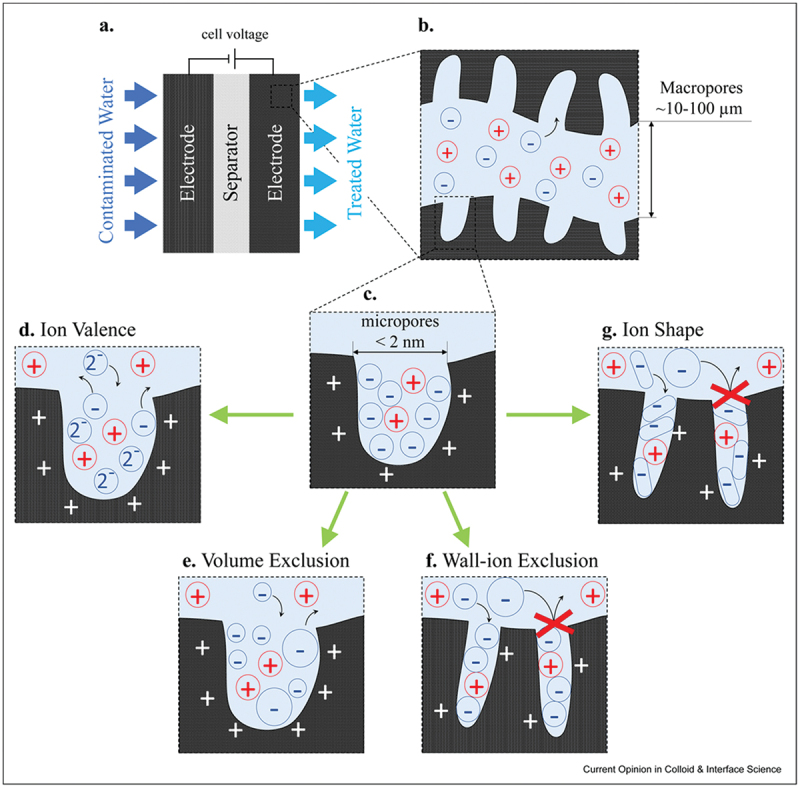
Schematic illustrating select ion-selectivity mechanisms by CDI for two competing anions. a. A CDI cell. b. A macropore that facilitates ion and water transport. c. A micropore enabling ion storage. d. Selectivity due to ion valence. e. Selectivity due to ion volume exclusion interactions. f. Selectivity due to wall – ion interactions. g. Selectivity due to ion shape [122]. Reproduced with permission obtained via copyright clearance center.

Shocron et al, extended numerical modeling of Electric Double Layer (EDL) formation by incorporating physical effects such as finite ion size, surface affinity, and dielectric corrections into the widely used Local Density Approximation (LDA) models [122]. Figure 11 illustrates how such models dissect the contributions of different mechanisms to the potential and selectivity profiles within micropores, and how these predictions align qualitatively with observed experimental selectivity trends.

Nordstrand et al. primarily focuses on numerical modeling through the use of ODEs [102]. The authors present a MATLAB-based tool that simplifies the numerical simulation process, allowing users to easily solve the governing equations for ion transport and electric double-layer formation. This tool provides a practical way to model CDI dynamics, offering valuable insights into optimizing the system’s performance under various operating conditions. More recently, Nordstrand et al. extended their numerical work by implementing a fully coupled, spatiotemporal 3D CDI model in COMSOL, highlighting how numerical modeling continues to evolve toward handling more complex geometries and operating conditions [107].

Each method provides unique insights and serves different purposes in understanding and optimizing CDI systems for water desalination.

#### Machine learning and data-driven optimization

Machine learning (ML) and data-driven approaches are transforming CDI research by introducing powerful tools for predicting performance, optimizing system variables, and guiding electron design. As highlighted by Olayiwola et al. [123], ML techniques such as data imputation, transfer learning, and metaheuristic optimization are helping overcome challenges associated with limited and incomplete datasets. These methods enable accurate prediction of salt adsorption capacities (SAC), identification of optimal operational parameters, and improved interpretation of structure-performance relationships. Frameworks such as ImputeNet and genetic algorithms have further facilitated integration of experimental and synthetic data streams, leading to better model generalizability. Collectively, these advancements underscore the transformative role of ML in improving the design, efficiency, and scalability of CDI systems.

To provide a comprehensive overview, the following subsections explore the application of ML in CDI across five key domains: predictive modeling, feature selection for material design, process optimization, integration with experimental workflows, and current limitations with future prospects:

##### Prediction of CDI performance

Several machine learning models have been developed to predict CDI performance metrics such as SAC, average salt adsorption rate (ASAR), and charge efficiency. Wang et al. (2024) applied random forest (RF), support vector regression (SVR), and artificial neural networks (ANN) to forecast SAC and ASAR based on features such as surface area, nitrogen content, and pore structure [12]. Among these, the random forest model demonstrated the highest predictive accuracy. These results demonstrate the feasibility of using ML as a rapid screening tool for evaluating CDI electrode performance.

##### Feature importance and electrode optimization

In addition to predicting CDI performance, machine learning models such as extreme gradient boosting (XGBoost) can help researchers understand which electrode features most strongly influence desalination efficiency. Hai et al. (2025) employed an XGBoost regressor to model the electrosorption capacity of activated carbon electrodes derived from palm kernel shells (PKSAC) synthesized under different activation conditions [124]. Their model successfully predicted the performance of various electrode samples, demonstrating good alignment with experimental results.

Although explicit feature importance ranking was not reported in their study, the integration of XGBoost modeling with experimental variation across porosity, conductivity, and activation chemistry implicitly highlights key factors driving performance. Their work illustrates how high-performance ML models can support electrode screening and guide experimental design, even in the absence of large datasets.

Future work combining XGBoost with explainability tools such as SHapley Additive exPlanations (SHAP) could further enhance our understanding of structure – performance relationships in CDI materials, enabling more targeted synthesis strategies [125].

##### Process parameter optimization

Machine learning has also been successfully integrated with traditional design of experiments (DOE) tools like response surface methodology (RSM) to fine-tune CDI operational parameters. For instance, Hai et al. (2025) developed a hybrid approach combining RSM and Extreme Gradient Boosting (XGBoost) to model and optimize parameters such as applied voltage, feed flow rate, NaCl concentration, and electrode spacing [124]. Their predictive model aligned well with experimental outcomes and enabled the identification of optimal operating conditions, such as 1.2 V, 7.5 mL/min flow rate, 750 mg/L NaCl concentration and electrode spacing of 3 cm, which led to a salt adsorption capacity of 22.5 mg/g. This approach not only reduced experimental workload but also improved system efficiency and provided a robust platform for scaling up CDI technology.

##### ML-Experiment integration and validation

Integration of machine learning (ML) models with experimental validation is essential for translating computational predictions into real-world applications. Integration of machine learning (ML) with experimental validation is critical for the real-world deployment of CDI technologies. In a comprehensive study, Wang et al. developed ML models Gradient Boosting (GB), Support Vector Machines (SVM), Artificial Neural Networks (ANN), and Random Forest (RF) to predict desalination performance based on six electrode features (e.g. specific surface area, pore volumes, nitrogen/oxygen content, graphitization degree) and three operational parameters (electrolyte concentration, applied voltage, flow rate [12]. Among these, the GB model outperformed others, achieving the lowest prediction errors for both salt adsorption capacity (SAC) and average salt adsorption rate (ASAR). To validate the ML predictions, the authors synthesized MOF-derived and biomass-derived porous carbons and used them in CDI experiments.

The close agreement between predicted and measured SAC/ASAR values confirmed the robustness of the ML approach and underscored its utility in guiding rational electrode design. This model-experiment synergy represents a significant step toward accelerating CDI development while minimizing trial-and-error experimentation.

##### Data limitations and emerging opportunities

One of the challenges in ML-based CDI research is the scarcity of large, standardized datasets. Olayiwola et al. emphasized the role of data augmentation and imputation tools such as ImputeNet to handle incomplete data and reduce bias [123]. Additionally, transfer learning [126] has emerged as a promising approach to generalize ML models across different CDI systems and operational domains despite limited data availability. Moreover, bibliometric mapping [127] offers an emerging opportunity to identify research gaps, prioritize data collection efforts, and guide interdisciplinary collaborations in CDI research. Future progress will depend on more open data-sharing, standardized benchmarking protocols, and closer collaboration between materials scientists and data engineers.

### Simulation supported by experiment

Simulation-supported experimentation plays a central role in bridging theoretical models with practical CDI system performance. Several recent studies have gone beyond theoretical modeling to validate simulation predictions with laboratory or pilot-scale experiments an approach critical for moving CDI from conceptual design to practical implementation. As computational techniques continue to evolve, validating these simulations through experimental studies ensures the reliability, scalability, and practical applicability of the findings.

**Figure 12.**
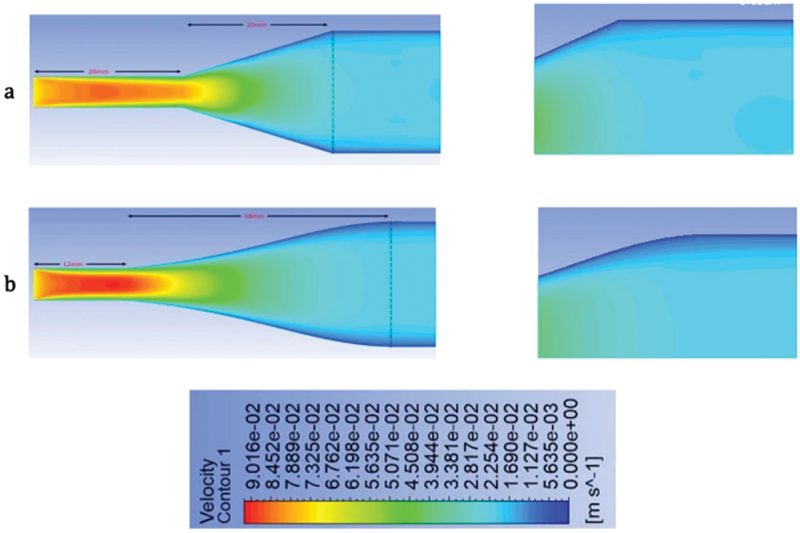
Flow velocity profiles of the initial reactor design (a) and the optimum design (b) [128], reproduced with permission obtained via copyright clearance center.

One notable study was published by Duranoğlu and Al-Aghbari, where CFD simulations were used to guide the design optimization of a CDI cell [128]. Their simulations guided the design modifications by predicting fluid dynamics and ion distribution, which aimed to enhance desalination efficiency by reducing dead zones and ensuring uniform flow. The CFD simulation using the ANSYS software showed that the optimized CDI design significantly reduced dead zones compared to the initial design (Figure 12), resulting in a more homogeneous velocity profile. Rounding the walls and corners of the flow channel generated laminar flow, enhancing the electrode’s effective area and maximizing salt removal capacity. The experimental results corroborated the simulation predictions, demonstrating a 47% improvement in performance, thus validating the effectiveness of simulation-driven design improvements. This integrative approach ensures that the CDI cell is both theoretically optimized and practically viable.

**Figure 13.**
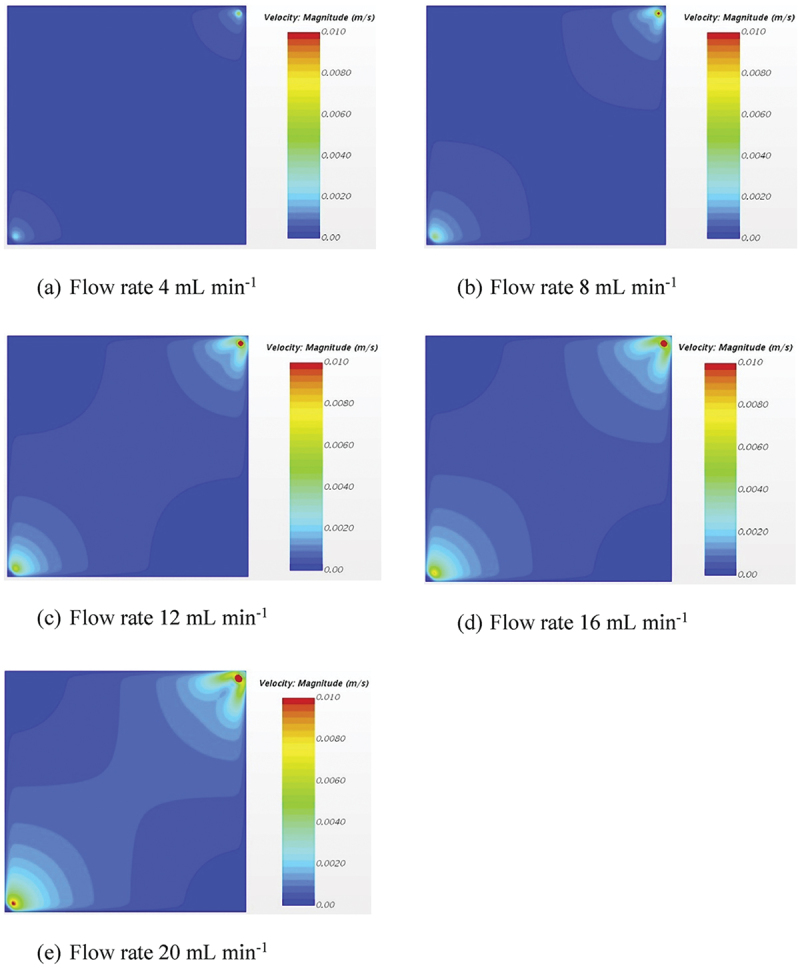
Velocity profile in square CDI channel at different flowrate, as predicted by CFD simulations [109]. Reproduced with permission obtained via copyright clearance center.

Another noteworthy example of simulation-validated experimentation is provided by Gaikwad et al. who demonstrated strong alignment between CFD predictions and experimental outcomes in a CDI system [109]. While their CFD analysis identified how increasing flow rate could reduce stagnant regions in a square CDI channel (Figure 13), the experimental data independently confirmed a significant enhancement in electrosorption performance using rice husk-derived activated carbon electrodes. The system achieved 85% Cr(VI) removal from a 10 mg L^−1^ feed, validating the CFD-predicted benefit of higher feed velocity. This study highlights the strength of simulation-experiment synergy in optimizing not just flow design, but also operating parameters that directly impact real-world performance.

**Figure 14.**
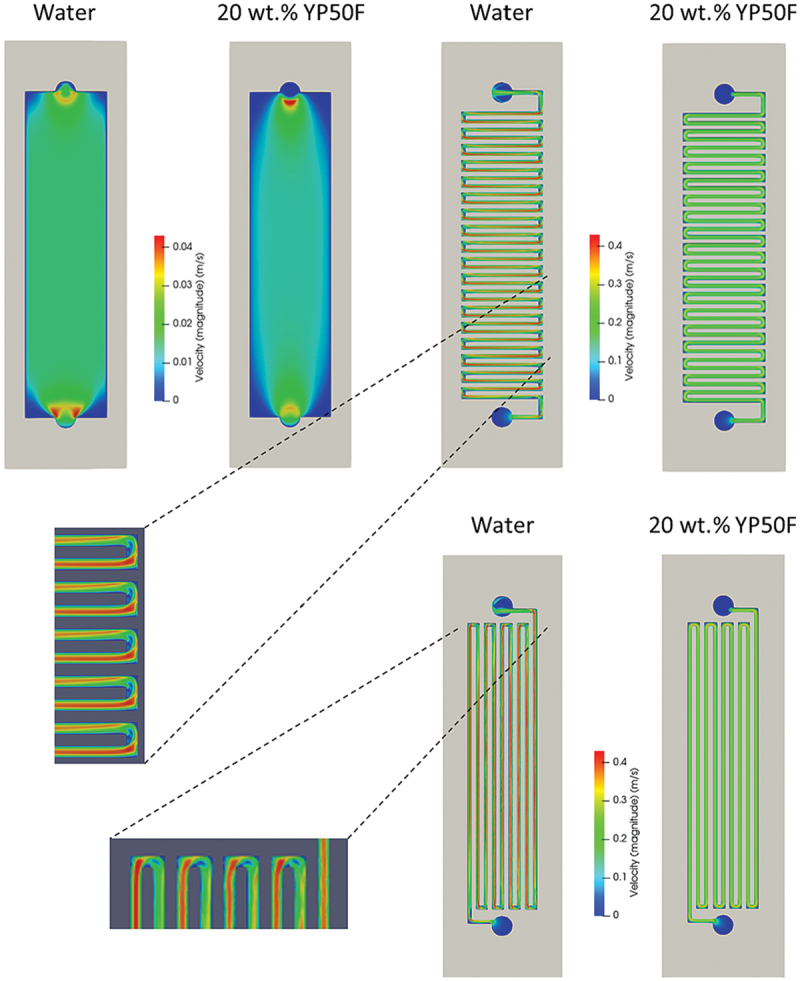
Slits (at the middle of the channel) of velocity fields (m/s) of water and flow electrode (20 wt% YP50F) in channels with open, serpentine vertical and serpentine horizontal geometries, as predicted by computational fluid dynamics (CFD) simulations [111]. Reproduced with permission obtained via copyright clearance center.

Similarly, Saif et al. conducted a comprehensive CFD and experimental study on Flow Capacitive Deionization (FCDI) to assess how channel geometry affects the performance of non-Newtonian flow electrodes [111]. Their simulations revealed that long, straight channels produced low local shear rates, increasing slurry viscosity and leading to clogging, especially in vertical serpentine designs. Conversely, horizontal serpentine channels induced velocity gradients that lowered viscosity and improved flowability. These findings from CFD were validated by experimental results, which confirmed that the horizontal serpentine configuration outperformed others in salt removal performance. The velocity field distributions shown in Figure 14 illustrate the differences in flow behavior between water and 20 wt% YP50F slurry across the three channel types. Despite improved flow conditions, the study also identified poor internal mixing as a limiting factor highlighting the need for new designs that better accommodate non-Newtonian behavior. This study exemplifies how simulation-experiment integration not only explains performance differences but also provides clear design guidance tailored to real-world flow electrode behavior in FCDI systems.

**Figure 15.**
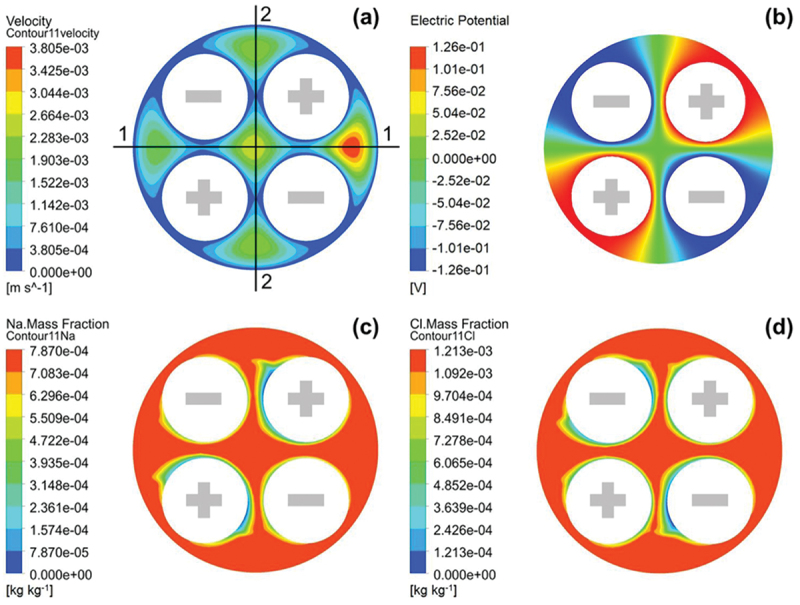
2D profiles for (a) flow velocity of the feed flow through the water channel, (b) potential field, (c) Na^+^ mass fraction, and (d) Cl^−^ mass fraction at the cross-sectional plane midway along the length of the FCDI apparatus, as generated by the CFD model for operation with 39.1 mL min^−1^ flow rate and 0.933 V applied voltage (corresponds with 0.20 a applied current) [110]. Reproduced with permission obtained via copyright clearance center.

He et al. (2024) further exemplified this synergy by developing and validating a 3D CFD model for a tubular-electrode-based FCDI system [110]. The model accurately predicted velocity distributions, electric potential fields, and ion concentrations under realistic operational conditions. Figure 15 presents 2D CFD simulation results showing the flow velocity (a), potential field (b), and mass fractions of Na^+^ (c) and Cl^−^ (d) ions in a cross-sectional plane. These results corresponded well with experimental performance metrics, confirming the model’s predictive accuracy. While the system achieved modest per-area salt removal performance, the expanded membrane area and net removal rate highlighted the effectiveness of the design. This work illustrates how validated simulations can inform the development of scalable and efficient CDI geometries by identifying performance-limiting regions and guiding further refinement.

Another compelling case is the use of a modified Poisson-Boltzmann equation by Usman et al. to investigate electro-adsorption mechanisms on conductive ultrafiltration membranes [51]. Their work highlighted how advanced theoretical modeling could elucidate ion interactions and adsorption efficiency at electrically charged surfaces, providing valuable insights into water treatment applications. The integration of such theoretical approaches with experimental validation emphasizes the importance of understanding the fundamental ion adsorption mechanisms to optimize the performance of CDI and related technologies, especially for hybrid CDI systems.

Additionally, Lim et al. developed a numerical model for FCDI that accounts for energy losses across membranes, electrodes, and flow paths [62]. This model was validated using pilot-scale experimental data, proving that theoretical predictions could accurately reflect real-world energy performance and desalination outcomes under varied salinity conditions.

**Figure 16.**
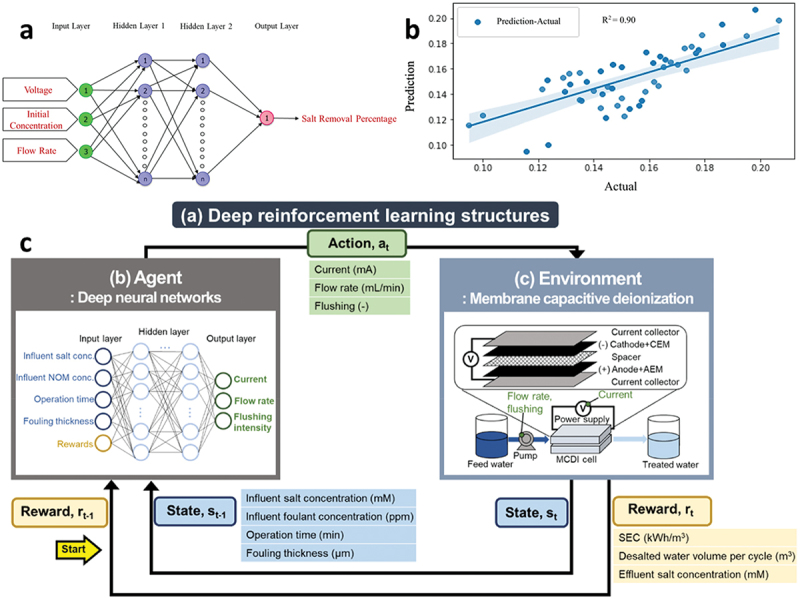
(a) Schematic process of predicting salt removal percentage via ANN model. (b) The comparison between the predicted data and actual data. (c) Schematic illustration of automating the MCDI process via DRL. Reproduced from [129]. Reproduced with permission obtained via copyright clearance center.

In addition to physics-based models, other researchers demonstrated how machine learning (ML) techniques can complement experimental CDI research by predicting desalination performance based on electrode properties and operational conditions [12]. In their study, four ML models were trained to predict salt adsorption capacity (SAC) and average salt adsorption rate (ASAR) of porous carbons, with the gradient boosting model achieving the highest accuracy (RMSE of 2.13 mg g^−1^ for SAC and 0.073 mg g^−1^ min^−1^ for ASAR). Feature analysis revealed that electrolyte concentration and specific surface area were the most influential factors. Experimental validation with metal – organic framework- and biomass-derived carbons confirmed the predictions, showcasing ML as a powerful tool to guide material and process optimization in CDI. The effectiveness of their ML-based approach is further illustrated in Figure 16, which shows the neural network architecture, prediction accuracy, and a deep reinforcement learning framework integrating experimental feedback for real-time CDI optimization [129].

Furthermore, Liu et al. [130] combined experimental studies with COMSOL-based CFD simulations to evaluate a novel CDI device designed for treating desulfurization wastewater. Their simulations revealed non-uniform flow distribution and dead zones at high flow rates, consistent with reduced desalination efficiency observed in experiments. At lower flow rates, the flow field was more uniform, improving overall performance. In addition to flow dynamics, they validated kinetic and isothermal adsorption models by fitting experimental data, showing that the pseudo-first-order kinetic model and the Freundlich/Redlich – Peterson isotherm models best described the ion adsorption behavior. These findings illustrate how combining COMSOL simulations with adsorption model validation deepens understanding of both fluid transport and electrostatic interactions in CDI systems.

**Figure 17.**
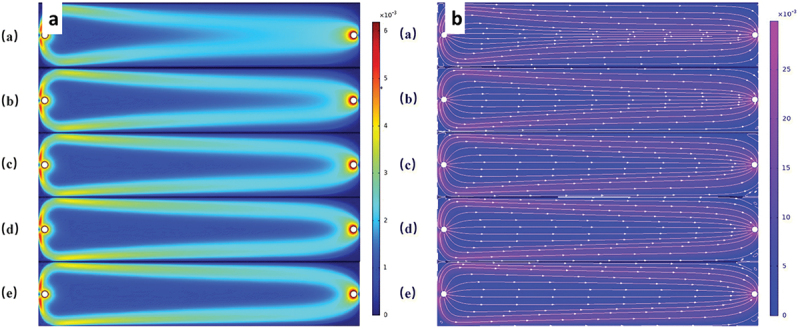
A) velocity profiles of rectangular CDI flow paths at different flow rates: (a) 100 mL/min, (b) 200 mL/min, (c) 300 mL/min, (d) 400 mL/min, and (e) 500 mL/min, b) streamlines profile of rectangular CDI flow paths at different flow rates: (a) 100 mL/min, (b) 200 mL/min, (c) 300 mL/min, (d) 400 mL/min, and (e) 500 mL/min [130]. Reproduced with permission obtained via copyright clearance center.

Figure 17 shows the simulated velocity profiles and streamlines of the rectangular CDI unit at different flow rates [130]. The simulated flow rate profiles complement the experimentally identified optimal operating conditions (voltage, pH, temperature) by ensuring that uniform flow and adequate residence time are achieved throughout the CDI cell, allowing the electrodes to fully exploit the enhanced electrosorption kinetics provided by those optimal conditions.

These diverse examples underscore the growing significance of simulation-experiment synergy in CDI development. Whether through CFD for fluid dynamics optimization, Poisson-based models for ion interaction analysis, or numerical simulations for energy efficiency evaluation, integrating modeling with experimental validation enables researchers to test hypotheses in silico and confirm them under realistic conditions. This approach not only strengthens the reliability of design choices but also accelerates system development, minimizes trial-and-error, and enhances confidence in scaling up CDI technologies for real-world applications.

## Emerging hybrid and coupled CDI technologies

Capacitive Deionization (CDI) has seen substantial advancement not only through electrode and reactor innovations but also by coupling it with complementary technologies. These hybrid systems aim to overcome CDI’s intrinsic limitations such as ion selectivity, fouling, or limited energy efficiency by integrating it with other desalination, purification, and energy-harvesting systems. Figure 17 summarizes various hybrid CDI configurations and their targeted applications such as contaminant degradation, selective ion removal, resource recovery, and energy efficiency [56].

### Hybrid electrodes and selective ion removal

Recent advancements have explored integrating CDI with materials or systems that enhance its ability to selectively target ions or degrade contaminants.

- Photocatalysis-CDI Hybrid Systems (PCS): By integrating photocatalysis and CDI, contaminants can be both degraded and removed in a single system. Photocatalysis handles the degradation, while CDI ensures ion removal, enhancing the treatment of both organic and inorganic pollutants [131].
- Ultrafiltration-CDI (UCDI): This hybrid combines ultrafiltration’s ability to remove organic pollutants with CDI’s ion removal capabilities, allowing for simultaneous deionization and organic matter removal while reducing membrane fouling [55].
- Ion-Exchange Coupling: CDI has been coupled with ion exchange membranes or resins to enhance selective ion removal, particularly for specific ions like nitrate or phosphate, which are otherwise poorly adsorbed by conventional carbon-based electrodes [132].

### CDI-Energy integration and recovery systems

Integrating CDI with energy-generating or energy-recovering systems offers a route to enhanced sustainability and reduced operational costs.

- Microbial Fuel Cells (MFC)-CDI Hybrid Systems: In this configuration, MFCs generate electricity through organic degradation, which is then used to power CDI for deionization. This integration allows for energy-efficient desalination while simultaneously treating wastewater [34,133].
- MCDC (Microbial Capacitive Deionization Cell): This extension of the MFC-CDI hybrid adds selective ion storage electrodes, improving energy recovery and contaminant separation efficiency [95].
- Photovoltaic and Thermally Regenerative CDI: Some systems utilize solar panels or thermally regenerative cycles to provide energy input for CDI, further reducing carbon footprints and enabling off-grid operation [134].

### CDI coupled with advanced water treatment processes

Beyond simple ion removal, CDI is being coupled with other advanced treatment processes for multi-functional performance.

- RO-CDI Hybrid Systems: Combining CDI with reverse osmosis (RO) helps in handling RO brine, improving water recovery and lowering energy consumption, making desalination more energy-efficient [37] RO removes bulk salts while CDI polishes the effluent or treats brine.
- FCDI-Membrane Hybrid Systems: Recent work by Lim et al. further underscores the potential for integrating CDI systems particularly flow-electrode CDI (FCDI) with membrane-based technologies [62]. Their simulation and pilot-scale testing revealed that FCDI can operate at lower energy costs than brackish water RO under low salinity and can handle higher salinity streams through membrane area optimization. The study also indicated that scaling membrane areas within FCDI can help manage higher salinity streams, suggesting a pathway for FCDI to be used either as a stand-alone system or as a hybrid module in conventional RO setups for enhanced energy and process efficiency.
- CDI-EO (Electrooxidation) and CDI-IE (Ion Exchange): Systems combining CDI with electrochemical oxidation enable concurrent removal and degradation of contaminants such as pharmaceuticals and dyes. Similarly, coupling with ion exchange processes allows CDI to act as a polishing unit for selective ionic species [19].

**Figure 18.**
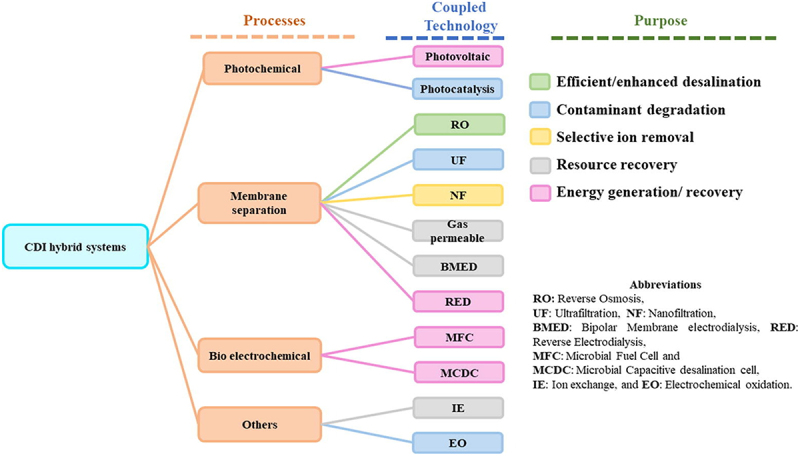
Flow chart illustrating various processes and coupled technologies with CDI technology for specific application/purpose. Different colour coding in the coupled technology column represents a separate application of the hybrid system based on colour codes given ahead of each purpose in the purpose column [56]. Reproduced with permission obtained via copyright clearance center.

These emerging hybrid configurations aim to enhance the flexibility, energy efficiency, and contaminant removal capabilities of CDI. They are tailored for specific applications from brine management and resource recovery to decentralized water purification and energy-neutral desalination. Additionally, a detail classification of CDI hybrid systems in Figure 18 [56] shows how CDI technology can be coupled with several processes and technologies. These include photochemical, membrane separation, ion exchange, electrochemical oxidation, and bioelectrochemical processes. Each hybrid system is designed with a specific purpose, such as contaminant degradation, resource recovery, or energy generation and recovery.

## Challenges and opportunities in CDI

Despite significant progress in CDI, several challenges remain that hinder its widespread application. These include both experimental and simulation-related limitations. This section presents a structured and integrated overview of these challenges and outlines potential pathways for addressing them. The challenges are also summarized in Table 2, which connects experimental and computational issues with key references and potential solutions.

**Table 2.** CDI challenges.

No	Challenge	Experimental	Simulation	Reference
1	Material cost	√		[7–9]
2	Performance		√	[8,102,135]
3	Electron transport		√	[8]
4	Early development stage		√	[8]
5	Up-Scaling and commercialization	√		[2,7,9]
6	Operational Complicity in managing high salinity water	√		[7,9]
7	Energy efficiency	√		[7,9]
8	Integration with other technologies	√		[7,9]
9	Material and configuration limitations	√		[9,57]
10	Fouling and scaling	√		[9,25]
11	Regeneration of electrode	√		[9,86]
12	Modeling complexity and accessibility		√	[102]
13	Flow rate and voltage dependence		√	[102]
14	Surface modifications impact		√	[102]
15	Generalization across different systems		√	[102]
16	Limited datasets		√	[129]

### Experimental challenges

#### Challenges in CDI

CDI has received growing attention due to its energy efficiency and low chemical consumption. However, its practical scalability and performance consistency face several hurdle [7,57,135]:

- Material costs and performance: Advanced materials like carbon nanotubes and activated carbon can improve adsorption capacity and rate, yet their high-cost limits large-scale implementation. A promising strategy is to use biomass-derived porous carbon or hydrothermally synthesized carbons, which reduce costs while maintaining reasonable performance.
- Energy efficiency: Although CDI is generally energy-efficient, improving energy recovery during desorption and minimizing energy losses remains challenging. Studies have highlighted the inefficiency of current systems from a thermodynamic perspective, which limits their scalability. Incorporating energy recovery modules, exploring novel charging strategies like constant voltage/current pulsing, and optimizing electrode capacitance can help enhance thermodynamic efficiency. Improvements can be achieved through energy recovery units, optimized charging-discharging modes, and system-level integration for energy reuse.
- Scalability and commercialization: Laboratory-scale CDI systems often show a decrease in efficiency when scaled up, primarily due to uneven flow distribution and increased internal resistance. Modular stack designs and optimized flow-field engineering are being explored to mitigate these effects and facilitate reliable performance at industrial scale.
- High salinity feedwater managements: CDI’s effectiveness decreases as the feed water salinity increases, requiring innovative solutions to maintain efficiency in high-salinity conditions. Hybrid CDI systems (e.g. CDI-RO, CDI-EO) and high-capacitance electrodes could extend CDI operation to high-salinity water or even seawater.
- Integration with other technologies (hybrid systems): Combining CDI with other processes (e.g. EO, UF, or advanced oxidation) can improve selectivity and removal efficiency, but introduces new challenges in system control and synchronization. Compact integrated units with defined functional separation may address these design complexities.

In summary, the experimental challenges in CDI are complex but addressable through strategic material design, system-level optimization, and hybrid integration approaches.

Overall, although CDI generally consumes less energy than conventional technologies, achieving high thermodynamic energy efficiency remains a major challenge. Most CDI systems experience intrinsic energy losses during the adsorption-desorption cycle, complicating fair comparisons with other technologies. Moreover, CDI performance is strongly influenced by electrode materials and cell configurations, underscoring the importance of continued research into material innovation, architecture optimization, and operational control to advance the practical applicability of CDI.

#### Challenges in FCDI

FCDI is an emerging variant of CDI which allows for the continuous operation. Despite its advantages, several challenges have been identified [8,17,31]:

- Material Cost: The conductive agents used to enhance the conductivity of flow-electrodes, such as carbon nanotubes and carbon black, are expensive. Adopting low-cost conductive additives (e.g. biochar, doped carbon) and surfactant-enhanced slurry formulations could lower material costs.
- Electron Transport: The performance of FCDI is significantly influenced by electron transport within the flow-electrodes. This transport is not only dependent on the conductivity of the solid electrode particles but also on their inter-particle connectivity. Optimizing particle size distribution, using conductive binders, and designing structured flow electrodes can improve conductivity and performance of FCDI systems.
- Early Development Stage: Despite its demonstrated advantages, such as continuous desalination and low energy consumption, FCDI technology is still in the early stages of development and is not yet ready for widespread practical application. Focus should be placed on long-term stability testing, up-scaling trials, and integration with real-world water metrics to support industrial application.

These challenges highlight the need for sustained research effort focused on improving the cost-effectiveness of materials and enhancing internal electron transport mechanisms. Addressing these limitations is essential to advance FCDI as a viable solution for large-scale, continuous desalination application.

#### Challenges in MCDI

MCDI enhances traditional CDI by incorporating ion-exchange membranes, which improves ion selectivity and efficiency. However, this technology also faces several challenges in experimental research [6,28,61,65,68,82]:

##### Membrane fouling

Biofouling and adsorption of organics reduce system efficiency and increases maintenance needs. Surface modification with developing antifouling coatings, dynamic surface charge control, and operational strategies like polarity reversal and appropriate pre-treatment can mitigate membrane fouling and improve membrane longevity.

##### High membrane costs and durability

Ion-exchange membranes increase system costs and may degrade under harsh conditions. Focus on recyclable membrane materials, UV-stabilized polymers, and crosslinked membranes could reduce cost and improve their lifespan and durability.

##### Energy efficiency and ion selectivity

Optimizing both energy efficiency and ion selectivity simultaneously remains difficult. Designing gradient membranes or layered structures with tailored ionic permeability and applying data-driven optimization can help balance selectivity and energy use.

##### Complex water matrices

MCDI systems face difficulties in treating water with mixed contaminants or high salinity. Selective electrode coatings and tailored membranes for multi-ion environments can help improve robustness and selectivity.

The Table 2 summarizes and categorizes CDI into specific areas such as material cost, performance, electron transport, up-scaling, and operational complexities. It distinguishes between experimental and simulation challenges, referencing various studies for each. This table reflects a structured approach to address the multifaceted challenges in advancing CDI technology through both experimental and computational strategies, as highlighted in the cited studies.

### Simulation challenges

Simulation models play a critical role in understanding and optimizing CDI systems. However, current models face limitations in accuracy, computational efficiency, and real-world applicability. The simulation of CDI systems also involves various complexities and challenges that can impact the data accuracy, efficiency, and applicability of models. These challenges can be categorized into several key areas as follow:

#### Model accuracy and complexity

- Simplifications and assumptions are often necessary in CDI modeling to make the problems trackable, but they can limit applicability of the results.

For example, simplified models such as the Donnan or dynamic Langmuir (DL) often neglect important real-life situation. While such assumptions help reduce computational time, they can sacrifice fidelity under variable conditions [136].

To address this trade-off, Advanced models have been developed that strike a better balance between realism and solvability. Notably, Nordstrand et al. [137] proposed a hybrid numerical-analytical model based on a 3D finite-element framework, and Haverkort et al. [108] introduced an analytical yet robust mathematical model. Both demonstrate how hybrid approaches combining analytical insights with numerical or data-driven techniques can improve predictive capability while maintaining numerical stability. These advances suggest that future CDI models will increasingly use hybrid approaches, which can stay computationally efficient while still accurately reflecting how systems behave in reality.

- To improve reliability, machine learning and data-driven calibration are increasingly applied to refine model parameters based on experimental data. Integrating such adaptive models with empirical observations helps mitigate the limitations of fixed-parameter systems.
- Parameter Estimation: Data variability and model sensitivity hinder predictive accuracy. Use of experimental databases and machine learning techniques for robust parameter extraction and uncertainty quantification.

#### Computational demands

- Computational Complexity: High-fidelity simulations, particularly those incorporating detailed physics or large-scale systems, can be computationally expensive. This includes the need for significant computational resources and time, which may limit the ability to perform extensive parameter sweeps or real-time simulations.

The 3D COMSOL model by Nordstrand et al. required approximately 100 minutes of computation time on a 24-core workstation, showing that while full-dimensional modeling is feasible, it still demands substantial resources. To overcome stability issues, they embedded their equations into existing COMSOL frameworks and applied techniques like anisotropic meshing and gradual voltage ramping to ensure convergence [107].

- Multi-Scale Challenges in Molecular Dynamics Simulations: MD simulations offer atomistic insights but often face limitations in modeling non-equilibrium processes relevant to CDI systems. Bridging the gap between simulated time and length scales and those observed experimentally is particularly challenging. For instance, a study by Liu et al. employed a two-scale model to simulate ion transport and adsorption in porous electrodes used for CDI [96]. While providing valuable insights, the study highlighted the difficulties in extrapolating MD results to experimental scales, underscoring the need for multi-scale modeling approaches to fully capture CDI processes. These challenges suggest that integrating MD simulations with continuum or mesoscale models may be necessary to comprehensively understand CDI processes, especially under non-equilibrium conditions.

- Scalability Issues: Up-scaling simulations from small lab-scale setups to larger, more practical systems can be difficult. This often requires adjustments in the model to account for scale-dependent factors such as changes in flow dynamics or electrochemical interactions, which can introduce additional sources of error.

Notably, the model by Nordstrand et al. [107] was designed to be flexible and geometry-independent, making it adaptable for exploring larger-scale or asymmetric CDI structures, including flow-through configurations with nonuniform inlet/outlet paths. This approach enhances the model’s scalability and potential for real-world applicability.

#### Integration with experimental data

- *Data Consistency*: Integrating simulation results with experimental data can be challenging due to discrepancies between the idealized model conditions and real-world experimental setups. Differences in cell geometry, electrode materials, and operational parameters can affect the comparability of simulation results with experimental data.
- *Validation and Calibration*: Ensuring that simulation models accurately reflect experimental conditions requires ongoing validation and calibration. This involves not only fitting models to experimental data but also understanding and addressing the sources of any discrepancies that arise.
- *Modeling Accuracy and Experimental Support*: Advanced theoretical frameworks, such as the modified Donnan local density approximation (LDA), have been improved to account for finite ion size, hydration energy, and wall – ion interactions, yet they often rely on fitted parameters without direct experimental corroboration. Shocron et al. highlighted how atomistic simulations can complement continuum models by capturing size effects, hydration energies, pore geometry, and ion orientation explicitly, offering a pathway to more physically meaningful predictions of ion selectivity in CDI [122]. However, systematic experimental validation of these atomistic insights remains limited, underscoring the need for closer integration between advanced modeling approaches and empirical measurements to build confidence in simulation-informed CDI design.

#### Generalization across different systems

- Material Variability: CDI systems often use a variety of electrode materials and configurations, each with unique properties. Creating a generalized model that accurately predicts performance across different materials and cell designs can be challenging, as it requires capturing the diverse behaviours and interactions of different system components.
- Operational Modes and Conditions: CDI systems can operate under various conditions, such as continuous or batch modes, and different flow configurations. Simulating these diverse operational scenarios while maintaining accuracy and computational efficiency requires robust models capable of adapting to different conditions.

Addressing these simulation challenges is crucial for improving the reliability and applicability of CDI models. By focusing on enhancing model accuracy, managing computational demands, ensuring consistency with experimental data, and generalizing across different systems, researchers can develop more effective and versatile tools for simulating CDI processes.

## Conclusion

Capacitive Deionization (CDI) represents a transformative approach to water desalination, particularly for low-salinity water sources, with notable advantages over conventional desalination techniques such as reverse osmosis and electrodialysis. Its energy efficiency, low environmental impact, and operational simplicity make CDI a compelling choice for brackish water treatment, resource recovery, and even environmental monitoring.

The integration of advanced materials, especially carbon-based electrodes such as graphene, carbon nanotubes, and carbon aerogels, has significantly enhanced CDI performance. Innovations like Membrane Capacitive Deionization (MCDI) and Flow-Electrode Capacitive Deionization (FCDI) further expand the capabilities of CDI, enabling more efficient ion removal and selectivity while also allowing for continuous operation in challenging conditions. These developments are poised to make CDI a valuable tool not only for water desalination but also for broader applications in energy storage, gas separation, and environmental remediation.

In addition to desalination, CDI holds significant promise in environmental monitoring. The technology’s ability to selectively remove specific ions from water makes it effective for tracking and mitigating pollutants in various water sources. By continuously monitoring ion concentrations, CDI systems can help detect contaminants such as heavy metals and nitrates, thus safeguarding water quality. Its low energy consumption and adaptability further enhance its utility in remote or resource-limited regions, supporting sustainable environmental protection efforts.

However, challenges persist. The cost of advanced electrode materials, the complexity of integrating CDI into large-scale systems, and the need for energy efficiency improvements in high-salinity conditions are key hurdles that must be addressed for widespread adoption. Simulation plays a critical role in addressing these issues, enabling optimization of reactor design, fluid dynamics, and material properties, thus providing invaluable insights that complement experimental efforts. By linking simulation with experimental validation, CDI systems can be fine-tuned to maximize efficiency and scalability, opening pathways for large-scale implementation.

Looking forward, impactful advancements in CDI will likely come from the integration of multifunctional electrodes that combine capacitive and Faradaic mechanisms to enhance charge efficiency, ion selectivity, and long-term operational stability. Materials such as heteroatom-doped carbons, MXene – carbon hybrids, phosphorus-doped bio-carbons, redox-active composites (e.g. WO₃-based hybrids), and Layered Double Hydroxides (LDHs) show promise in bridging the gap between high performance and durability. At the system level, coupling CDI with renewable energy sources (such as photovoltaic or thermally regenerative inputs) and advanced treatment technologies (e.g. electrooxidation or ultrafiltration) could drive modular, energy-efficient solutions. Finally, predictive simulation – experiment feedback loops, using DFT and multiscale modeling, are essential for accelerating the rational design of electrode materials and optimizing ion transport, ultimately guiding CDI toward scalable and application-specific deployment. In conclusion, CDI holds immense potential as a sustainable, low-energy desalination technology that can address global water challenges. Continued innovations in materials, reactor design, and simulation methods will be essential to realizing the full potential of CDI in practical, large-scale applications, ultimately contributing to global efforts to ensure water security and environmental sustainability.
